# Evaluation of the school-based ‘PhunkyFoods’ intervention: a cluster randomised controlled trial in the UK

**DOI:** 10.1017/S1368980025000552

**Published:** 2025-04-14

**Authors:** Karen L. Vaughan, Milca Vidal, Janet E. Cade, Marion M. Hetherington, Charlotte E.L. Evans

**Affiliations:** 1 School of Food Science and Nutrition, University of Leeds, Woodhouse Lane, Leeds LS2 9JT, UK; 2 School of Psychology, University of Leeds, Woodhouse Lane, Leeds LS2 9JT, UK; 3 Purple Nutrition, 17 Hazel Drive, Chesterfield S40 3EN, UK

**Keywords:** Cluster-RCT, Food Literacy, Cooking skills, Complex intervention, Fruit and vegetable intake, Healthy eating

## Abstract

**Objective:**

We evaluated the impact of an established nutrition education intervention, ‘PhunkyFoods’ on food literacy, cooking skills and fruit and vegetable intake in primary school aged children.

**Design:**

A pre-registered cluster randomised controlled trial was used; the intervention group received the ‘PhunkyFoods’ programme and the wait-list control group received the usual school curriculum. Primary outcomes measured were differences in food literacy and cooking skills scores between the intervention and control arms after 12 months adjusted for baseline values.

**Setting:**

The trial was undertaken in twenty-six primary schools in North Yorkshire, UK.

**Participants:**

631 children aged 6–9 years participated (intervention *n* 307, control *n* 324) through assemblies, classroom activities and after-school clubs.

**Results:**

There were no significant effects of the intervention compared with control on food literacy, cooking skills, vegetable intake or fruit intake. Adjusting for baseline, the Food Literacy Total Score was 1·13 points lower in the intervention group than the control (95 % CI –2·87, 0·62, *P* = 0·2). The Cooking Skills Total Score was 0·86 lower in the intervention group compared with the control (95 % CI = –5·17, 3·45, *P* = 0·69). Girls scored 2·8 points higher than boys in cooking skills across the sample (95 % CI = 0·88, 4·82, *P* < 0·01).

**Conclusion:**

The intervention did not result in improved food literacy or cooking skills, though sex effects on these outcomes were observed. More practical food preparation hours are needed in primary schools to improve the likelihood of an effect on outcomes.

Acquiring competent food preparation and cooking skills is an important part of children’s education that can lead to improved healthy lifestyles and dietary behaviours^(1,2)^. Studies have shown that school-based cooking interventions which have experiential learning activities have the potential to increase food literacy knowledge, cooking skills and fruit and vegetable intake^(3–5)^. Recent research has shown an association between food literacy and vegetable intake in adolescents^(6)^. Experiential learning with sufficient hours of practical food preparation activities can increase vital skills such as chopping, weighing, mashing, measuring and cooking on the hob^(3,7)^. It is through learning these practical food preparation techniques that primary school aged children can show improved cooking self-efficacy and also small but significant changes in food intake, especially vegetable intake^(5)^. Previous research has found that child learners of cooking skills have better outcomes than teen learners or adult learners, suggesting that if these skills are learned at a younger age, then individuals are more likely to identify themselves as cooks^(8)^.

Since children spend so much time in school each week, this is a useful place to acquire the skills and knowledge related to healthy diets and healthy lifestyles. The Nutrition-Friendly Schools Initiative was set up in 2006 outlining twenty-six essential criteria within five components, including nutrition and health-promoting curricula although there are no essential criteria specifically related to developing food preparation skills in children^(1)^. Policy analysis of the food curriculum in eleven countries undertaken by Smith *et al.* in 2022 describes approaches as either practical (Home Economics) or health oriented (Health and Physical Education). The authors developed a framework to evaluate ‘Food Preparation Skills’ in each country, assessing the contribution to food literacy within the curriculum^(9)^. Their analysis revealed that whilst countries often have a mandatory food curriculum (and would therefore meet the essential criteria for a ‘Nutrition-Friendly School’ as defined by the WHO), there is still ‘no consensus in primary food education’ about what this includes and more specifically, if it includes the teaching of food preparation skills such as chopping, grating and mashing^(9)^. These are essential life skills that can be learned in stages through the early school years, to improve children’s habits and diets, since using food preparation techniques to cook a meal from scratch usually involves eating more fresh fruit and vegetables^(7,10,11)^.

The importance of interventions designed to improve the food education curriculum in schools is a priority for public health. Good nutrition and maintaining a healthy weight in childhood helps to prevent obesity and diet-related ill health later in life^(1,4,12,13)^. However, data from the National Child Measurement Programme in the UK show that the prevalence of children living with obesity more than doubled from 9 % at the start of school to around 22 % at age 11 years^(14)^ so better nutrition education is needed to improve food skills and food literacy in childhood.

This research is the first fully powered efficacy cluster randomised controlled trial to evaluate the impact of a lifestyle intervention programme on food literacy, cooking skills and fruit and vegetable intake for primary-aged children aged 7–9 years in the UK. PhunkyFoods is an established multicomponent intervention which has been delivered in the UK for over 20 years and the design has evolved using the COM-B model of behaviour change^(15)^. Research perspectives in this evaluation focus on effectiveness and theory-based approaches to increase knowledge and understanding of what works^(16)^.

## Objectives

A cluster randomisation approach was chosen for the practical reasons of recruiting schools and is more ecologically valid for conducting interventions with schools. The main aim of the cluster randomised controlled trial was to assess the impact of the PhunkyFoods healthy lifestyle intervention programme on food literacy, cooking skills and fruit and vegetable intake of children compared with the usual practice in primary schools. For the fourth hypothesis, we originally included an index of multiple deprivations as a potential mediator in the trial protocol. However, this was removed on reflection, since it was felt that percentage eligibility for free school meals (%FSM) was a better indicator for deprivation and both were not needed. The research hypotheses as outlined in the trial protocol are as follows^(17)^:

1. The PhunkyFoods intervention group will show higher food literacy and cooking skills than the control group measured by mean scores.
2. The PhunkyFoods intervention group will show a higher intake of fruit and vegetables than the control group measured by mean scores.
3. Schools in the intervention arm that choose more ‘active ingredients’ (intervention components) from the flexible menu of PhunkyFoods options will have better outcomes than those schools that choose less ‘active ingredients.’
4. The school-level measure of deprivation (%FSM) will have a mediating impact on the outcomes for schools in the intervention arm.

## Methods

### Trial design

The study was a parallel, cluster randomised controlled trial, with two arms and with a 1:1 allocation ratio. The unit of the cluster was the school. The intervention arm received the PhunkyFoods programme, with introductory training from May 2022, and the wait-list control arm received the PhunkyFoods programme after the final research data collection was completed, with introductory training from May 2023. This was a superiority trial, designed to test if the intervention was more effective than the control. The trial was registered prospectively with ISRCTN (ISRCTN68114155) in October 2021, and the study protocol was published in 2022^(17)^. The study was informed by a feasibility pilot study from 2019^(18)^. The authors followed the CONSORT reporting guidelines for cluster randomised trials^(19)^.

### Sampling and participants

#### School recruitment

Power calculations determined that thirteen clusters (schools) were needed in each arm to detect a moderate effect size with 90 % power, based on a previous study by Dean *et al.*, 2021^(20)^. This calculation assumed that a mean of fifteen children would be included in each school, and therefore 195 would be needed in each arm. Only one class per school participated in the research. Due to the clustered nature of the two-level model where individual children are clustered in schools, a conservative estimate of 20 % variation at the school level was assumed.

Recruitment was from December 2021 to March 2022. Inclusion criteria for eligible primary schools in Harrogate or Selby in the North of England included having at least one class with twenty or more students aged between 7 and 9 years. Seventy-four eligible schools were invited to participate in the study using invitation letters, adverts in North Yorkshire Council communications, Huntington Research school newsletters and social media. Follow-up contacts were made to schools using telephone calls and emails to headteachers. Headteachers who expressed an interest were invited to attend a short 20-minute online school briefing to inform them about the PhunkyFoods programme and the requirements of the research project. Eligibility criteria were amended in February 2022 to allow three rural schools to participate with smaller numbers of children (*n* 9, *n* 11, *n* 14) to improve recruitment numbers. The online school briefings were delivered by the lead researcher (KV) with support from a member of the PhunkyFoods team. Schools were offered one full year of fully funded PhunkyFoods intervention at their school if they participated in the research project. It was explained that some participating schools would receive the intervention in the first year (intervention group) and that some schools would receive the intervention in the second year (wait-list control group). The PhunkyFoods programme has been well established in the UK and has been running for 20 years. This likely impacted recruitment success.

### Participant recruitment

Once the headteacher of the school had signed a school consent form, we requested a 15-minute telephone meeting to complete the enrolment to the project. This meeting was to identify the research class (participants) and main contact person for the school. After the research class was identified, a parent information letter was sent to the parents of the research class with an option to opt out of the study.

### Intervention

**Figure 1.**
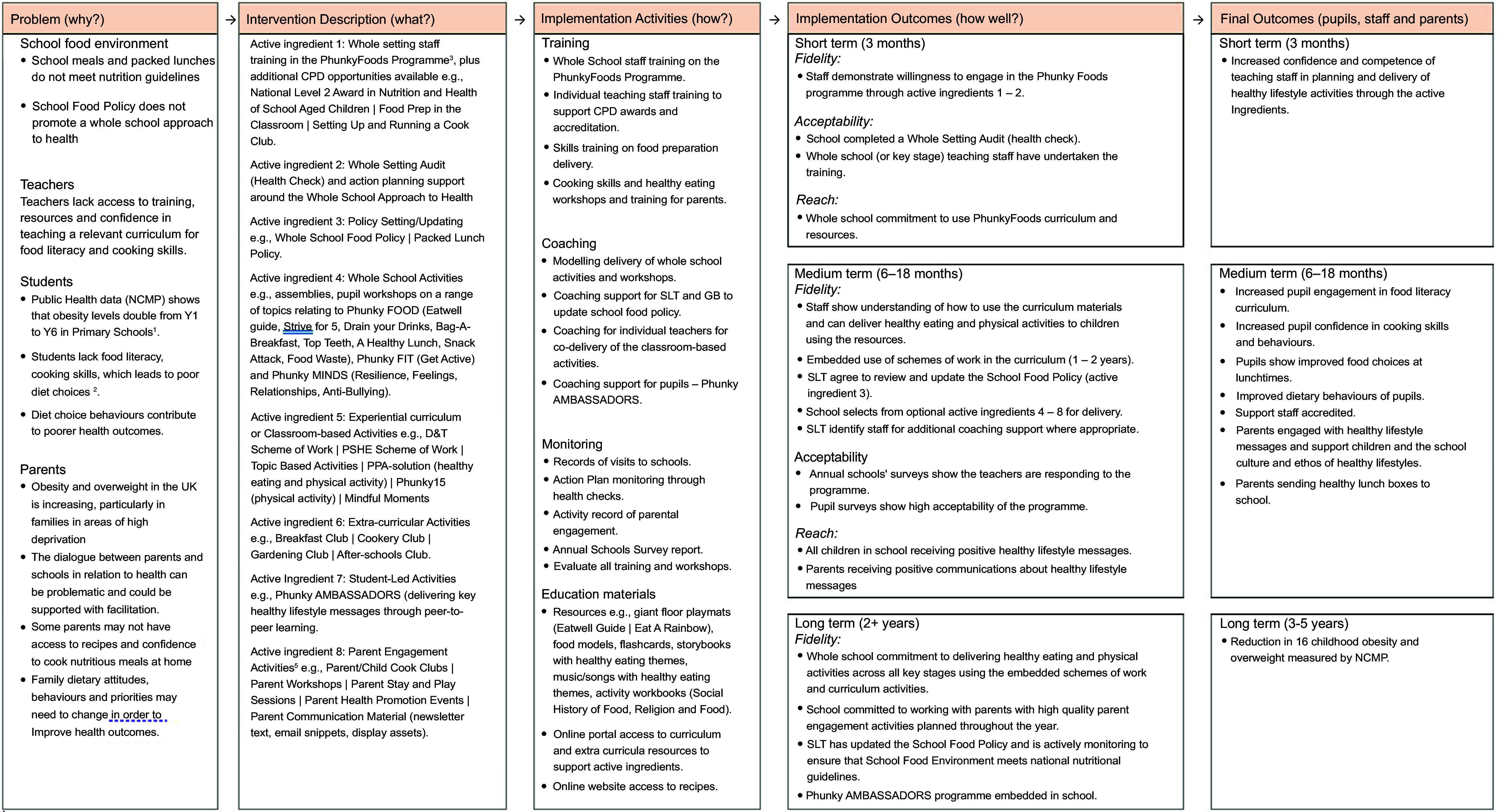
Logic model for PhunkyFoods intervention. ^1^NHS DIGITAL. 2020. *National Child Measurement Programme, England 2019/20 School Year* [Online]. Available: https://digital.nhs.uk/data-and-information/publications/statistical/national-child-measurement-programme/2019-20-school-year [Accessed]. ^2^DIMBLEBY, H. 2021. National Food Strategy: The Plan. UK. ^3^SAHOTA, P., CHRISTIAN, M., DAY, R. & COCKS, K. 2019. The feasibility and acceptability of a primary school-based programme targeting diet and physical activity: the PhunkyFoods Programme. *Pilot Feasibility Stud,* 5, 152. ^4^CHARLTON, K., COMERFORD, T., DEAVIN, N. & WALTON, K. 2020. Characteristics of successful primary school-based experiential nutrition programmes: a systematic literature review. *Public Health Nutr*, 1–21. ^5^AXFORD, N., BERRY, V., LLOYD, J., MOORE, D., ROGERS, M., HURST, A., BLOCKLEY, K., DURKIN, AND MINTON, J. 2019. How Can Schools Support Parents’ Engagement in their Children’s Learning? Evidence from Research and Practice. London: Education Endowment Foundation.

The intervention contains a flexible menu of eight ‘active ingredients’ (programme optional components), which are detailed in the PhunkyFoods Logic Model in Figure 1. PhunkyFoods has a detailed Delivery Manual with implementation guidance and resources for education and development coordinators (EDC) to deliver each of the eight active ingredients in partnership with schools. The initial introduction and training for school staff about the PhunkyFoods intervention took place from April to July 2022. The delivery period of the intervention to children was from September 2022, and this was for 5–7 months until follow-up.

It was expected that all schools would demonstrate a willingness to engage in the programme by completing the first two ‘active ingredients’ of staff training in the summer term of 2022. The first involved whole-setting staff training in the PhunkyFoods Programme with additional continuous professional development opportunities available for schools to opt into. Examples of continuous professional development opportunities proposed included the English Northern Council for Further Education (NCFE) National Level 2 Award in Nutrition and Health of School Aged Children^(21)^; Food Preparation in the Classroom and Setting Up and Running a Cook Club. Following the staff training was a health check on policy involving a whole setting audit and action planning support. This consisted of an initial meeting with a member of the senior leadership team to work through the Health Check document, then co-production of an action plan for development priorities agreed.

Activities with pupils started in September 2022. The Phunky Ambassadors programme involved mentoring pupils to deliver key healthy lifestyle messages through peer-to-peer learning. The PhunkyFoods intervention has a vast collection of resources available for schools to access, some of which are available to all on the website and more curriculum planning resources for members using a school login^(22)^. Whole school assemblies were delivered by the pupils on the Phunky Ambassadors programme in year 5, aged 9–10 years. Classroom activities were delivered to smaller groups of children and most often involved the Phunky Ambassadors. Schools were also invited to start up a cooking club, after-school cook club and parent engagement programme.

### Outcome measurements

Primary outcome measures were Food Literacy and Cooking Skills. Food literacy is measured by the Tool for Food Literacy Assessment in Children (TFLAC-UK) at baseline and at 12 months. The original questionnaire was developed by Amin *et al.* in 2019,^(23)^ and the UK version is available in Appendix 1. Food literacy is scored from 0 to 40, with 40 being the highest score indicating better food literacy. Cooking skills were measured by CooC11 at baseline and 12 months^(20)^. Cooking skills are scored from 0 to 55, with 55 being the highest score indicating better cooking skills. The survey is available in Appendix 3.

Secondary outcome measures were fruit intake and vegetable intake measured using a shortened version of the Child Assessment of Diet Evaluation Tool (CADET) at baseline and 12 months^(24)^. The food diary is available in Appendix 2. This tool is intended to be used retrospectively as a tick-list record for all foods consumed over a 24-hour period. In this case, we just selected fruits and vegetables from the larger tool which aimed to collect data on all foods consumed during a day. In total, the consumption of thirteen fruits and eighteen vegetables and pulses was asked in five mealtimes (breakfast at home, lunch at school, before tea, evening meal and after tea). The dietary information was analysed on the website ‘nutritools’^(25)^ using the Food Questionnaire Creator pages. The portion sizes used in CADET vary by age (3–11 years) and gender and are based on National Diet and Health Survey mean weighted consumption data^(26)^.

### Dose, reach and fidelity of intervention

The PhunkyFoods programme is a flexible menu of component ingredients, where schools can choose how much they want to engage. Whilst the Logic Model shows how fidelity to the intervention components can lead to improved health outcomes, the practical approach to delivery is deliberately flexible to encourage signups from schools. This aspect of real-world research poses a methodological challenge, in that not all the clusters (schools) get the same treatment in the intervention. To address this aspect, information was collected from each of the intervention clusters on how much of the programme they engaged with by the EDC employed by Purely Nutrition Ltd, who had delivered the programme. A single EDC assigned an overall engagement score from 0 to 14 for the schools that they worked with, based on their experience and knowledge of which components the school engaged with. Scores for each component were 0–2, depending on how many activities were delivered and if they were led by the EDC or the school, as shown in Table 3.

### Randomisation

The allocation to trial arms was undertaken using a computer-generated randomisation sequence. Block randomisation was used to ensure equivalence between the intervention and control schools for the geographical area (Selby or Harrogate), size of school (above or below the median school size) and above or below the median for % free school meals eligibility. The lead researcher (KV) enrolled participants and allocated unique ID numbers. After baseline data collection, one member of the research team (CE), who was blinded to the names of schools enrolled, generated the allocation sequence. The lead researcher then informed schools which trial arm they were in.

### Data collection

Data collection events were standardised across all schools and completed at two-time points. Data were collected at baseline and follow-up (March 2022 and March 2023, respectively) during school hours. Only one visit per school was allocated, and so children absent on the day of collection were not included. During the data collection visits, children completed the Food Literacy Survey (40 min) and the Cooking Skills Survey (15 min). These were completed as a whole class activity, with a member of the research team reading out each question to the class. For the CADET food diaries, one member of the research team introduced the research tool to the class, checking their understanding of a diary by asking the pupils questions and then reading out the instructions on how to complete it. Children were asked to take the tool home and bring the completed CADET food diary back to school the next day. The research team then collected the returned CADET food diaries over the following three weeks, sometimes returning to schools more than once to collect as many food diaries as possible.

### Statistical methods

The statistical analysis plan has been published previously as part of the trial protocol^(17)^. In summary, the primary and secondary analysis for the evaluation of PhunkyFoods intervention focused on two research perspectives (effectiveness and theory-based) identified in the framework for developing and evaluating complex interventions Medical Research Council guidance update^(16)^. Our analysis was conducted on an ‘intention-to-treat’ basis, in that all pupils with a recorded outcome at baseline and follow-up were included. We used SPSS software (IBM SPSS Statistics for Windows, Version 29.0.2.0 Armonk, NY: IBM Corp, released 2024) and allowed for the design effect of cluster trials in schools with pre–post design using multi-level regression models, using child-level covariates at level 1 and school-level covariates at level 2^(27)^.

## Results

A total of thirty-four schools expressed an interest in starting the trial and attended one of the online school briefings. Of these, six were excluded due to geographical eligibility and one was excluded as it had previously had some involvement with the PhunkyFoods programme. Sixteen 20-minute online Schools Briefings were delivered between December 2021 and March 2022. In all, twenty-seven schools completed baseline data collection but one school dropped out of the trial immediately after randomisation due to lack of school capacity.

Of the participating North Yorkshire schools, thirteen of these were in Harrogate and thirteen were in Selby. Baseline characteristics of the schools and pupils are shown in Table 1. The median value for percentage of free school meals in both groups was 10·7 % compared with the national median of 24·6 %^(28)^. Whilst the control and intervention groups looked similar in their characteristics, the exception to this was sex. A chi-square test showed a significantly higher proportion of females in the control group (58 %) compared with the intervention group (42 %), χ^2^ = 5·45, *P* = 0·02.

**Table 1.** School and child characteristics for the intervention and control groups at baseline

	Control	Intervention	Total
School characteristics			
Schools	13	13	26
Geographical location of schools			
Harrogate	7	6	13
Selby	6	7	13
Size of school (number of students)			
Median	195	170	192
IQR	105–257	121–201	105–212
Size of research class (number of students)			
Median	29	24	27
IQR	25–32	22–30	22–30
School level % free school meals			
Median	10·7	10·7	10·7
IQR	6·2–21·5	7–18·5	6·5–21·5
Child characteristics			
Number of participating pupils	324	307	631
Age in years			
Median	8·4	8·4	8·4
IQR	8–9·1	7·9–8·9	7·9–9
Sex			
Male	141	172	313
Female	183	135	318

**Figure 2.**
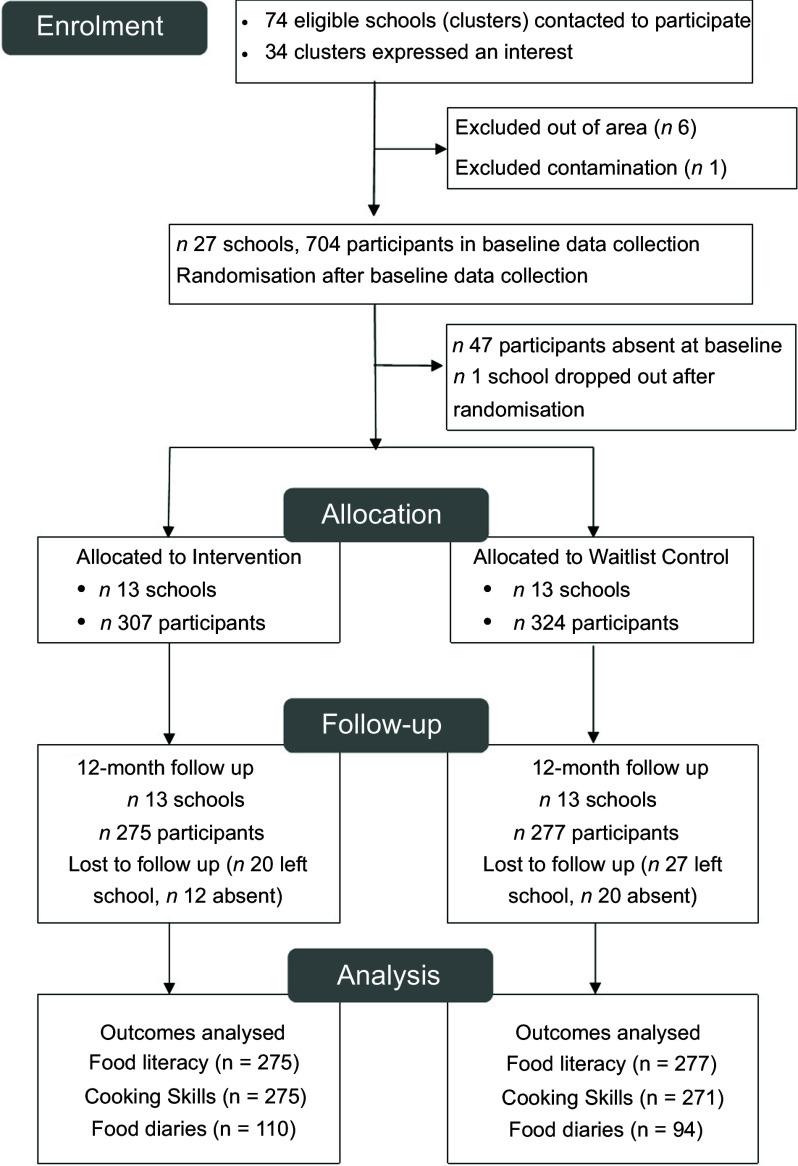
Consort flow diagram.

The number of participants recruited and who completed baseline data collection was 704 (Figure 2). However, since one school dropped out immediately after baseline data collection, the number who completed the trial was 307 children assigned to the intervention arm (in 13 schools) and 324 children in the control arm (in 13 schools). At 12-month follow-up, 552 pupils were present, of which 275 were in the intervention arm and 277 in the control arm. The Food Literacy and Cooking Skills surveys were completed in the classroom with the researchers.

The CADET diaries were completed by participants partially in the lesson at school and completed at home and returned to school the next day. We excluded nine extreme cases from the CADET diary analysis by using SPSS to compute the following criteria: if the vegetable frequency variable was greater than 16 and/or the fruit frequency variable score was greater than 18. An inspection of the excluded extreme cases showed an incorrect understanding of the survey; for example, the participant ticked all the fruit and vegetable boxes for every meal. The number of CADET diaries included in the analysis was 110 for the intervention group and 94 for the control group. See Figure 2.

### Intervention delivery

Appendix 4 shows detailed information on the number of schools that participated in each of the ‘active ingredients’ of the programme along with some examples of activities delivered.

### Food literacy and cooking skills

The between treatment group difference for Food Literacy Total Score was –1·13 (95 % CI –2·87, 0·62, *P* = 0·2), using a mixed multi-level analysis of covariance (ANCOVA) model with adjustment for baseline. Control group scored 1·13 more than the intervention group but this was not statistically significant. Food literacy domain-specific Cronbach alpha values were cooking skills 0·53, cooking knowledge 0·40, nutrition knowledge 0·67, food systems knowledge 0·35 and self-efficacy regarding eating 0·73. Although some of the scores seemed low, this is not surprising since dichotomous items have little variability and many of the survey questions were scored 0 or 1. Food literacy scores were calculated out of a total of forty and were significantly higher at follow-up than baseline in both the control and intervention groups. At baseline, scores across all the food literacy domains (cooking skills, cooking knowledge, nutrition knowledge, food systems knowledge and self-efficacy around eating) were similar for control and intervention groups. See Table 2.

**Table 2.** Differences in mean scores on food literacy, cooking skills, vegetable intake and fruit intake

Outcome	Intervention				Control				Between-group differences				Between-group differences			
									(1–0) minimally adjusted^§^				(1–0) fully adjusted^||^			
	Pre mean	sd^*^	Post mean	sd^*^	Pre mean	sd^*^	Post mean	sd^*^	EMM	*P*-value	95 % CI	ICC	EMM	*P*-value	95 % CI	ICC
Food literacy survey																
Cooking skills	2·3	0·6	2·4	0·6	2·3	0·6	2·5	0·5	−0·03	0·55	−0·14, 0·07	0·02	0·00^¶^	0·98	−0·19, 0·19	0·00^¶^
Cooking knowledge	4·7	1·2	5·1	1·1	4·8	1·1	5·2	1	−0·08	0·46	−0·31, 0·14	0·04	−0·14	0·58	−0·64, 0·36	0·04
Nutrition knowledge	11	2·5	11·7	2·4	11·2	2·5	11·9	2·3	−0·08	0·72	−0·52, 0·37	0·02	−0·62	0·1	−1·36, 0·13	0·00
Food systems knowledge	9·5	2	10·1	2	9·7	2·3	10·3	1·9	−0·13	0·49	−0·5, 0·25	0·02	−0·51	0·22	−1·36, 0·33	0·02
Self-efficacy	3·3	1	3·5	0·8	3·5	0·8	3·6	0·7	−0·06	0·38	−0·21, 0·09	0·02	−0·01	0·95	−0·35, 0·32	0·03
Food literacy total score^†^	30·9	5·2	32·8	4·7	31·5	5·1	33·5	4·6	−0·27	0·53	−1·12, 0·59	0·04	−1·13	0·2	−2·87, 0·62	0·03
Cooking skills survey																
Number of skills	6·6	3	7	2·9	6·8	3	7·2	2·9	−0·12	0·64	−0·57, 0·36	0·01	−0·22	0·64	−1·15, 0·71	0·00^¶^
Cooking skills total score^†^	25·7	14	27·4	13·7	27·1	14·7	29	14·3	−0·89	0·4	−2·93, 1·21	0·00^¶^	−0·86	0·69	−5·17, 3·45	0·00^¶^
CADET food diaries																
Vegetable intake^‡^ (g)	187	96	175	125	213	111	195	126	−11·64	0·5	−45·67, 22·39	0·00^¶^	−52·36	0·14	−122·75, 18·03	0·00^¶^
Fruit intake^‡^ (g)	313	217	373	273	342	257	410	312	−24·31	0·54	−106·88, 58·25	0·00^¶^	−79·21	0·34	−250·34, 91·92	0·01

ICC, intraclass correlation coefficient; EMM, estimated marginal means.

* Raw mean score (sd).

† Primary outcome.

‡ Secondary outcome.

§ EMM minimally adjusted for clustering and baseline.

|| EMM fully adjusted for clustering, baseline and covariates (sex, % eligibility for free school meals, school engagement score).

¶ Value is less than 0·005.

The between treatment group difference for Cooking Skills Total Score was –0·86 (95 % CI = –5·17, 3·45, *P* = 0·69) using a mixed multi-level ANCOVA model with adjustment for baseline. The control group scored 0·86 more than the intervention group controlling for baseline, but this was not statistically significant. The internal consistency reliability for cooking skills was high with a Cronbach’s alpha score of 0·85. Cooking Skills Total Score was out of a total of 55 and was also significantly higher at follow-up than baseline in both the control and intervention groups. At baseline, the most frequent of the eleven surveyed skills used in the control and intervention groups were mixing, weighing and chopping.

### Fruit and vegetable intake

Fruit and vegetable portion scores over 24 hours from the CADET diaries showed no statistically significant differences between treatment groups. The difference between the treatment groups was –52·36 g for vegetable intake portion score (95 % CI = –122·75, 18·03, *P* = 0·14), using a mixed multi-level ANCOVA model with adjustment for baseline. The control group scored 52 g higher than the intervention group, but the difference was not statistically significant. The difference between the treatment groups was –79·21 g for fruit intake portion score (95 % CI = –250·34, 91·92, *P* = 0·34), using a mixed multi-level ANCOVA model with adjustment for baseline. The control group scored 80 g higher than the intervention group but this was not statistically significant.

### Secondary analysis – mediating impact of covariates

The secondary analysis shows the mediating impact of school engagement level, a school-level measure of deprivation (percent of pupils eligible for free school meals) and sex of participants.

Contrary to our hypothesis, the results showed no difference between schools that chose more ‘active ingredients’ from the flexible menu of options in the PhunkyFoods programme than those schools that chose fewer ‘active ingredients.’ In Table 3, the change in means from baseline to follow-up was colour-coded to reflect if these were higher than the average change in means for the intervention group. School 10, with the highest engagement score of 13 out of a maximum of 14, showed a positive change of 3·0 for cooking skills and 2·8 for food literacy, which was higher than the intervention group average of 1·6 for cooking skills and 1·9 for food literacy. School 8 had an above-average engagement score of 10 and had the greatest increase in cooking skills of 8·6 and an increase in food literacy of 3·9. However, school 18 had the lower score on engagement, with only 1 point for attending initial training and yet scored 4·9 higher in cooking skills from baseline to follow-up. Interestingly, it was noted that one school had an average engagement score of 8, but both cooking skills and food literacy scores were lower at follow-up than at baseline. This school had fourteen pupils who received the intervention, thirteen of whom were boys.

**Table 3.**
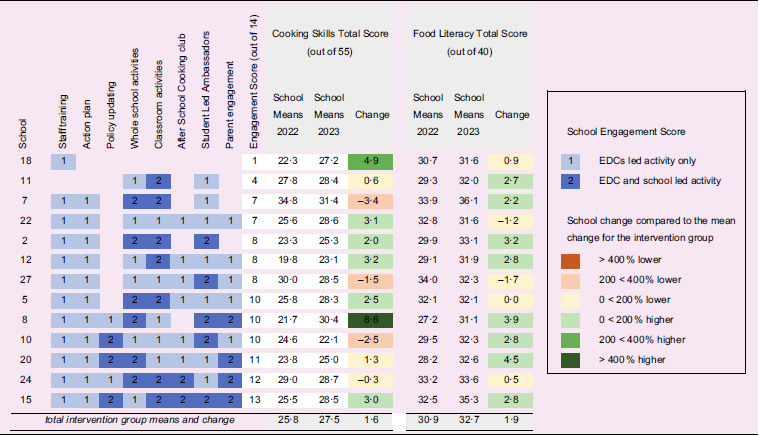
Effects of school engagement on primary outcome scores for intervention schools

EDC, Education Development Coordinator.

**Figure 3.**
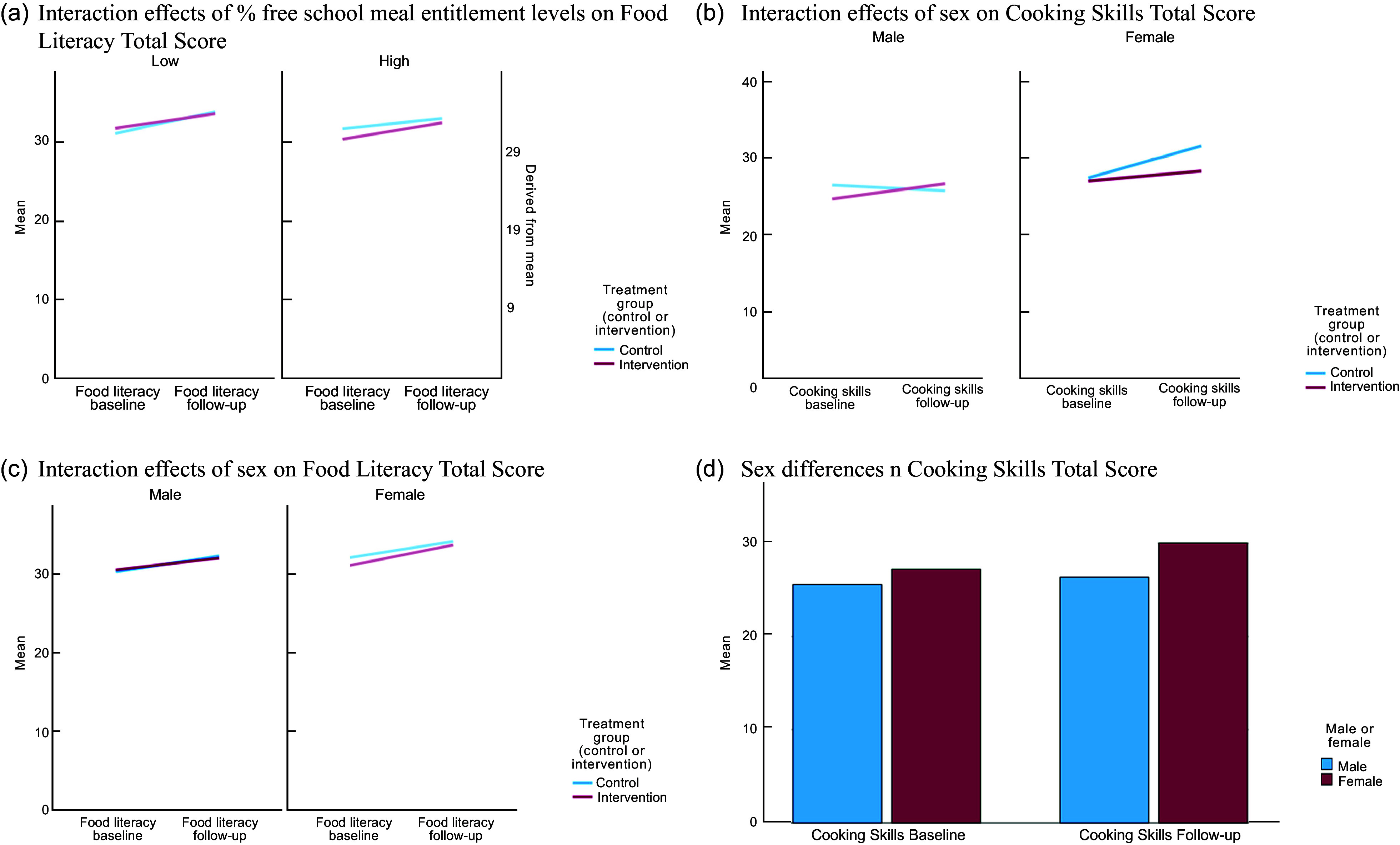
Secondary analysis: a) interaction effects of % free school meal entitlement levels on food literacy, b) interaction effects of sex on cooking skills, c) interaction effects of sex on food literacy and d) sex differences in cooking skills.

To explore the mediating impact of % free school meals, the level of free school meals was grouped into high or low in relation to the median for the sample. The results showed that the intervention had a very small (–0·05) but statistically significant effect, with a greater impact on food literacy for schools with a higher % of free school meals than the control group (95 % CI = –0·085, –0·011, *P* = 0·013). See Figure 3.

Due to sex differences between the intervention and control groups found in the baseline characteristics (Table 1), further exploratory analysis was conducted (this was additional to the pre-specified analysis). Using a multi-level regression model, the analysis showed a significant fixed effect of sex on food literacy scores, with girls scoring 1 point higher than boys (95 % CI = 0·4, 1·63, *P* = 0·001) across the total sample. See Figure 3. There was a statistically significant, fixed effect of sex on cooking skills, with girls scoring 2·8 points higher than boys (95 % CI = 0·88, 4·82, *P* > 0·01).

## Discussion

Overall, the results showed no substantial impact of the PhunkyFoods intervention on food literacy, cooking skills, fruit intake or vegetable intake. When examining individual school engagement, those schools with higher engagement in the intervention did not have greater improvements in food literacy or cooking skills than schools with lower engagement. Schools with a higher level of % free school meals showed a slightly higher but significant change in food literacy scores from baseline to follow-up in the intervention group compared with the control group.

Previous studies of cooking interventions have shown a positive impact on food literacy outcomes^(5)^. In North Carolina, Hovland *et al.*, measured food literacy using food-based science knowledge questions (with a total score out of 13) and showed a raw mean difference of 1·21 favouring the intervention (no 95 % CI reported)^(29)^. In Alabama, Parmer *et al.* measured food literacy using a nutrition knowledge survey with sixteen items (including food groups, nutrient knowledge, fruit and vegetable identification) and results showed a 1·37 raw mean difference (no 95 % CI reported) favouring the intervention^(30)^. However, it is important to note the difference in length and dose of the food preparation and cooking ‘treatment’ of these interventions compared with the current study. The Hovland study included 24× 45-minute food science lessons, and the Parmer study was a 14-hour programme. In comparison, many of the PhunkyFoods intervention schools had just 4 h of food preparation or cooking activities over 7 months. It is suggested that more intensive cooking interventions may be more effective.

The impact of lifestyle intervention programmes on cooking self-efficacy is often very small, even with longer intervention periods. A meta-analysis of six studies involving cooking lessons in primary schools showed a standardised mean difference of 0·39 (95 % CI = 0·05, 0·74) for cooking self-efficacy in children aged 4–12 years^(5)^. The Project CHEF study in Vancouver, Canada showed a standardised mean difference of 1·6 (95 % CI = 1·12, 2·08) favouring the intervention and included 15 h of cooking^(31)^. This study, evaluated by Zahr *et al.*, showed the largest impact on cooking self-efficacy, and it is relevant to note that the participating students had four or five sessions (two and a half hours long) where children learned knife skills and made eleven recipes from scratch.

The limited effectiveness of previous cooking interventions on cooking self-efficacy, food literacy and vegetable intake suggests that it is important to investigate what types of school-based approaches and improvements can be made^(3,5,32)^. The design of the PhunkyFoods programme is intentionally flexible, in the hope that this will encourage schools to sign up and participate at a pace appropriate for the individual school context. The varying levels of engagement in the programme can be seen in Table 3, ranging from a score of 1 to a score of 13, out of a total 14. Feedback from schools that have participated in PhunkyFoods is extremely positive, indicating that this flexible approach meets their variable and changing capacity needs well^(33)^. Nutrition education programmes whose mission is to work collaboratively with schools, arguably need to be flexible to foster long-term effective partnerships on policy and curriculum reform. From this perspective, complex interventions are not only understood from the number of different components but also from the varying relationship dynamics and school contexts in each setting and how flexibility helps to develop trust and longer-term commitment to change.

There is a further flexibility within each component of the programme, which may further influence impact. One example is the after-school cook club component, which although was designed to be at least 6 weeks in duration, in reality, lasted 4 weeks, due to difficulties in negotiating a commitment to the full 6 weeks. The cooking clubs usually only had between 6 and 12 pupils participating, and often this meant that only 50 %, or less, of the research class was involved. In addition, some schools opted to have different families attending the after-school cook club each week, which extends the reach but dilutes the treatment effect for each participant even further. It is recommended that at last 6 h of cooking is needed for whole class groups rather than small after-school clubs for some children.

The tension between model fidelity and flexibility in real-world complex interventions may account for why the trial results did not show an association between school engagement and outcomes. Implementation science theory concepts highlight these challenges, in particular the interplay of drivers such as capability, opportunity and motivations of schools to engage and the reach, effectiveness and adoption of programme elements^(15,34,35)^.

Since there were more girls in the control group than in the intervention group (and girls are known to have higher cooking skills than boys), the analysis of the data also explored the impact of sex differences. A recent study by Labbe (2023) involving primary school children showed that girls scored better in cooking skills, food knowledge and food skills in both the control and the intervention groups^(36)^. An evaluation of the ‘Cooking With Kids’ programme by Cunningham-Sabo *et al.* (2014) found that males made twice the gains in cooking self-efficacy compared with females^(37)^. However, the analysis in this study did not find an interaction effect between sex and treatment. Further analysis of the sex differences with the age variable confirmed that this effect was not due to the girls being older than boys.

It is likely that the number of food preparation and cooking hours for each research participant was insufficient to make an impact on cooking self-efficacy and fruit and vegetable intake. A recent systematic review showed that 6 or more hours of cooking are needed to make a difference in cooking skills and vegetable intake^(5)^. Of the seven schools that participated in the cooking club, it has already been noted that one school had different children attending each week, so the treatment dose was in total just 1 h of cooking. Studies involving only 1–2 h of cooking lessons have previously shown no impact on outcomes for the intervention group compared with the control group^(38,39)^.

We noted that the mean scores for vegetable and fruit intake (Table 2), using the CADET shortened survey, were much higher than other studies and exceeded the recommended combined 400 g of fruit and vegetable guidelines by the WHO^(40)^ and contrast sharply with findings from the WHO Child Obesity Surveillance Initiative survey, which found that only 45 % of children eat fruit daily and only 25 % eat vegetables^(41)^. In comparison, a study using the whole CADET tool, Project Tomato in the UK, showed baseline scores of 110 g for vegetables and 195 g for fruit^(42)^.

### Strengths and limitations

The main strength of this study was the robust cluster randomised controlled trial design, which was informed by a previous feasibility trial to improve the timeline for data collection of baseline measures before randomisation and the sample size was properly powered to detect an effect. The study was supported by a steering group with members of North Yorkshire Council and the Huntington Research school, who actively engaged with practical support around communication with schools and recruitment^(43,44)^. This enabled us to recruit a sample of twenty-six schools that completed the trial and increased the power of the statistical analysis.

The main limitation of the study is the representativeness of the sample to the whole of the English population. The median eligibility for free school meals for this study is 10·7 % but the median for primary schools in England is 24·6 %. Due to the flexible design of the intervention, model fidelity was very poor and we know that the delivery of the treatment will have varied considerably. Although we collected some information on school engagement, this was not extensive and was based on the assessment by EDC on their perception of how much schools engaged, which is subjective and relies on memory. A more realistic measure of school engagement could have been to check the number of times that each intervention school accessed the school resources on the PhunkyFoods website, but this was not possible with the resources available.

It is possible that the shortened format of the CADET food diary to assess fruit and vegetable intake may have inadvertently led to over-reporting. The CADET was validated for children aged 8–11 years against a weighed food method, maintaining its reliability and validity for nutrient analysis in children’s diets^(45)^. However, Christian *et al.*, 2015 discussed that CADET diaries tend to record higher fruit and vegetable intakes compared with weighed records, likely due to participants overestimating portion sizes^(45)^. Additionally, the National Diet and Health Survey age-related portion size data used for analysing CADET, despite being comprehensive, sometimes relies on relatively small sample sizes for specific foods, potentially leading to overestimations.

Social desirability bias is another likely factor influencing the high intake reports. The presence of researchers in classrooms to introduce and explain the diaries might have prompted children to report higher consumption of fruits and vegetables, aligning with perceived expectations. This bias is well-documented in dietary assessments where participants, particularly children, may alter their responses to conform to social norms or perceived preferences of the researchers^(46)^.

A final limitation concerns the short duration and low dose of the intervention in this trial. The PhunkyFoods programme is designed to build relationships with schools for curriculum change with an increased dose of the intervention components over time. It is possible that the intended outcomes of increased food literacy and cooking skills of children (as shown in the Logic Model) could have been achieved with a longer duration of 18 months or 2 years for the current trial.

## Conclusions

Robust research evidence is important to understand how to improve cooking skills, food literacy and dietary intake in children, which can help to improve the design and impact of healthy lifestyle interventions in schools. This research strengthens the evidence on the effectiveness of cooking interventions in schools by adding to knowledge about what works and in what circumstances. The PhunkyFoods intervention is a flexible whole-school approach to developing a health-promoting school, and the dose of food preparation and cooking skills within the programme may not be sufficient to show an impact on the outcomes. It is possible that some of the PhunkyFoods components that are easier to deliver and more popular with schools are less effective on food literacy and cooking skills outcomes than the more practical food preparation components. It is recommended that the programme could consider how to increase the number of experiential food preparation and cooking hours and the number of children participating in these activities to help increase the likelihood of producing significant benefits to children sooner. Further research is needed to explore sex differences in cooking skills and food literacy, and if there is an association between cooking skills, food literacy and obesity in this age group in the UK and other countries.

## Supporting information

Vaughan et al. supplementary material 1 Vaughan et al. supplementary material

Vaughan et al. supplementary material 2 Vaughan et al. supplementary material

Vaughan et al. supplementary material 3 Vaughan et al. supplementary material

Vaughan et al. supplementary material 4 Vaughan et al. supplementary material

Vaughan et al. supplementary material 5 Vaughan et al. supplementary material

## Supplementary material

For supplementary material accompanying this paper visit https://doi.org/10.1017/S1368980025000552
