# Supplementary material for: Evaluation of the school-based ‘PhunkyFoods’ intervention: a cluster randomised controlled trial in the UK
Source: Public Health Nutr. 2025 Apr 14;28(1):e86. doi: 10.1017/S1368980025000552 (PMC12100561; doi:10.1017/S1368980025000552)
Supplement: Vaughan et al. supplementary material 1 — Vaughan et al. supplementary material [file S1368980025000552sup001.docx]

Key Stage 2 Food Literacy Survey

This survey is part of a research project with Phunky Foods and the University of Leeds. The questions are based on academic research about Food Literacy^[[1]](#footnote-1)^. We want to find out about food literacy for children at Primary Schools. No individual names will be used as part of our research. We anonymise the data using individual numbers instead of names so that no person can be identified.

| Please put a tick in the box if you are happy for this survey to be used for research. ☺ |  |
| --- | --- |

**About you**

| Name of your school: |  | | |
| --- | --- | --- | --- |
|  |  | | |
| Your name: |  | | |
|  |  | | |
|  | Day | Month | Year |
| What is your date of birth? |  |  |  |
|  |  | | |
| What school year are you in now? |  | | |
|  |  |  |  |
| What is today’s date? | Day | Month | Year |
|  |  |  |  |

**Food Literacy Survey: Student Instructions**

Hello,

Thank you for agreeing to complete the Food Literacy Survey!

**What is Food Literacy?**

Food literacy is the knowledge and skills you use every day to make choices about food. This includes cooking, gardening, eating, and more.

**How to Take this Survey**

Read each question and mark the answer that you think is correct. If you have a question, please raise your hand.

**After the Survey**

There are 34 questions in the survey. After you are finished turn your survey back to the front page facing up and wait for further instructions.

**Questions #1-3: There are many skills needed to cook and prepare the food we eat every day. Please circle the answer based on whether you can do each skill “all by yourself”, “with a little help”, “with a lot of help”, or “not at all”.**

1. **I can use a knife to cut up food into smaller pieces, like cutting a cucumber or carrot into slices**
2. All by myself
3. With a little help
4. With a lot of help
5. Not at all
6. **I can crack an egg into a bowl**
7. All by myself
8. With a little help
9. With a lot of help
10. Not at all
11. **I can use a measuring jug to measure ingredients, like milk or flour**
12. All by myself
13. With a little help
14. With a lot of help
15. Not at all

**Questions #4-6: Which kitchen tool is used for each activity? Please circle the kitchen tool that matches each description.**

**On Page 5, you will find pictures of each kitchen tool to help you answer these questions.**

1. **Picks up hot food safely.**
2. Oven glove
3. Chopping board
4. Peeler
5. I don’t know
6. **Removes skin from fruit or vegetable.**
7. Spoon
8. Spatula
9. Peeler
10. I don’t know
11. **Rinses food and drains liquid from solid food.**
12. Peeler
13. Colander or strainer
14. Measuring jug
15. I don’t know

***Pictures of kitchen tools to help you answer questions #4-6.***

| **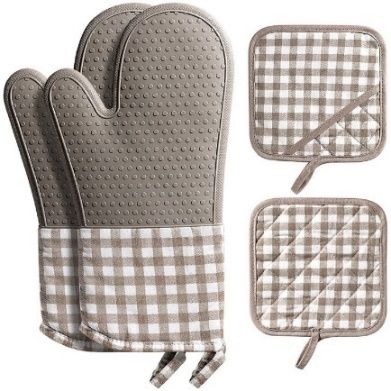** |  | **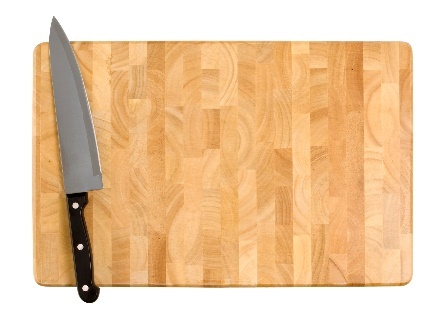** |  | **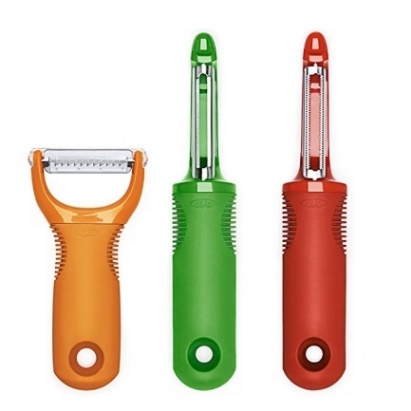** |
| --- | --- | --- | --- | --- |
|  |  |  |  |  |
| **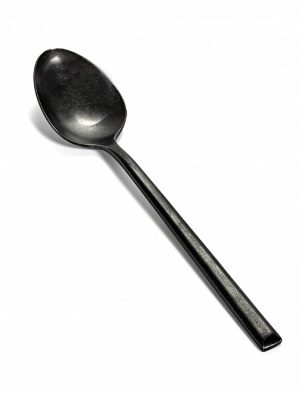** |  | **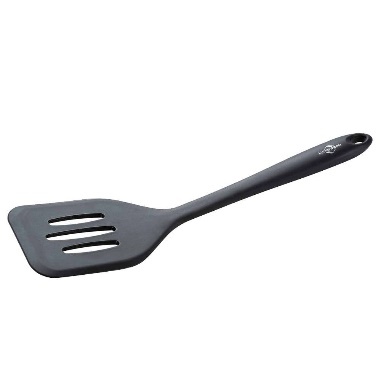** |  | **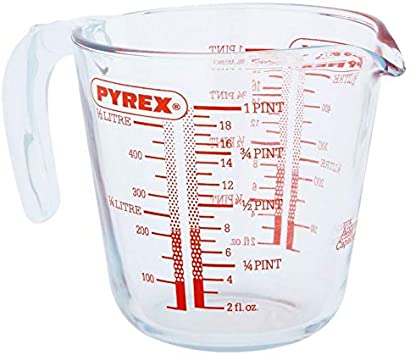** |
|  |  |  |  |  |
|  |  | **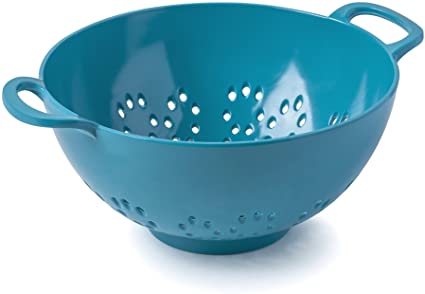** |  |  |
|  |  |  |  |  |

**Food safety is how we protect the foods we eat every day from germs, like bacteria and viruses, that can make people sick.**

**Please answer questions #7-9 about Food Safety:**

1. **To make sure that the foods we eat are safe, which of the following should be clean before cooking? Circle all the items below that should be clean before cooking.**
2. Hands
3. Chopping board
4. Floor
5. Cooking utensils (like a spatula or wooden spoon)
6. I don’t know
7. **Some foods are only safe to eat if they have been cooked first. Circle all the foods below that should be cooked first before eating.**
8. Oranges
9. Raw chicken
10. Carrots
11. Cheese
12. Eggs
13. I don’t know
14. **Circle True or False:**

**When preparing meals, raw meats (like raw hamburger) should not touch other foods (like salad or fruit) until the raw meat has been cooked.**

**True OR False**

1. **You are choosing foods to put in your lunch bag or on your lunch tray. For the main meal and two side dishes, circle the option (a OR b) that would make the healthiest meal.**

**Main Meal (circle a OR b)**

| 1. Pepperoni pizza | OR | 1. Turkey sandwich on brown bread |
| --- | --- | --- |

**Side Dish 1 (circle a OR b)**

| 1. Sweet potato fries | OR | 1. Broccoli |
| --- | --- | --- |

**Side Dish 2 (circle a OR b)**

| 1. Apple slices | OR | 1. Chocolate pudding |
| --- | --- | --- |

1. **Oscar is a Year 6 student who would like to buy a snack from the shop near his school before he gets on the bus at the end of the day. Which snack would be the healthiest for Oscar to choose? (circle ONE)**
2. Cheese and onion crisps
3. Oatmeal raisin cookie
4. Carrot sticks
5. Crackers
6. I don’t know
7. **An adult is taking you to a restaurant for dinner and asks you what you would like to drink. Which would be the healthiest drink to order (circle ONE)**
8. Water
9. Fizzy drink (like Coke or Sprite)
10. Diet fizzy drink (like Diet Coke or Sprite Zero)
11. Lemonade
12. I don’t know
13. **Which of the following things are not needed for a fruit or vegetable to grow?**
14. Soil
15. Sunlight
16. Water
17. Garden tools
18. I don’t know

**Questions #14-16: The vegetables we eat every day come from different parts of vegetable plants.**

**Draw a line to match each vegetable to the plant part it comes from.**

| 1. **Broccoli**   **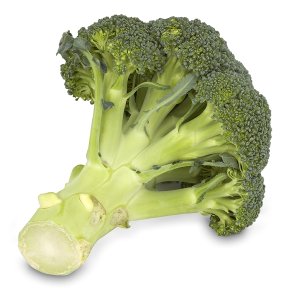** | **Root** |
| --- | --- |
| 1. **Carrot**   **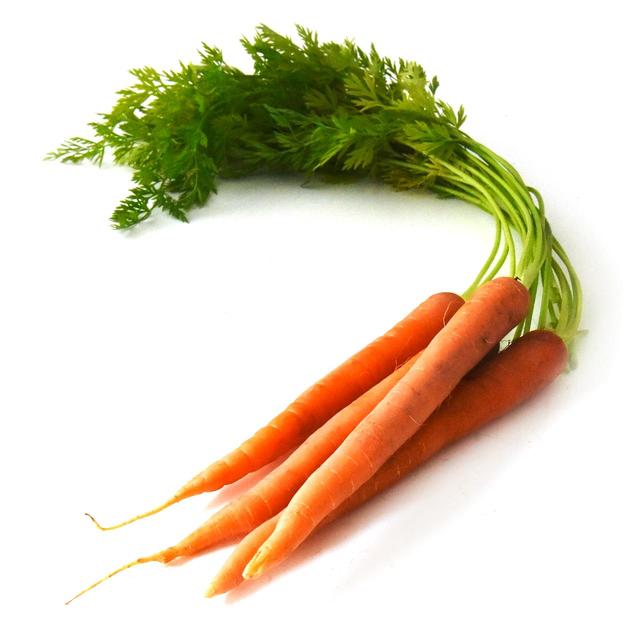** | **Leaves** |
| 1. **Spinach**   **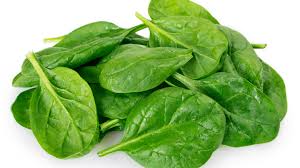** | **Flowers** |

**Questions #17-19: Some foods come from animals.**

**Draw a line to match each food listed below to the animal that it comes from.**

| 1. **Honey**   **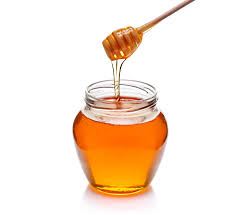** | **Pigs**  **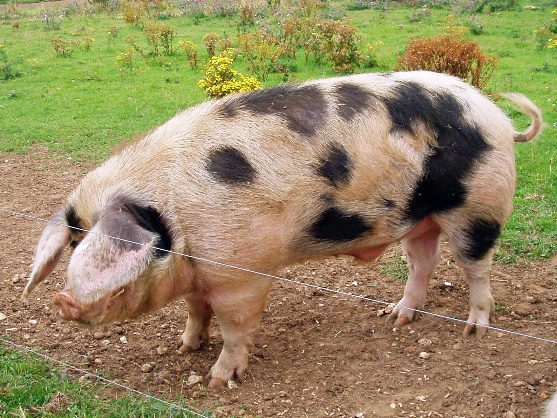** |
| --- | --- |
| 1. **Cheese**   **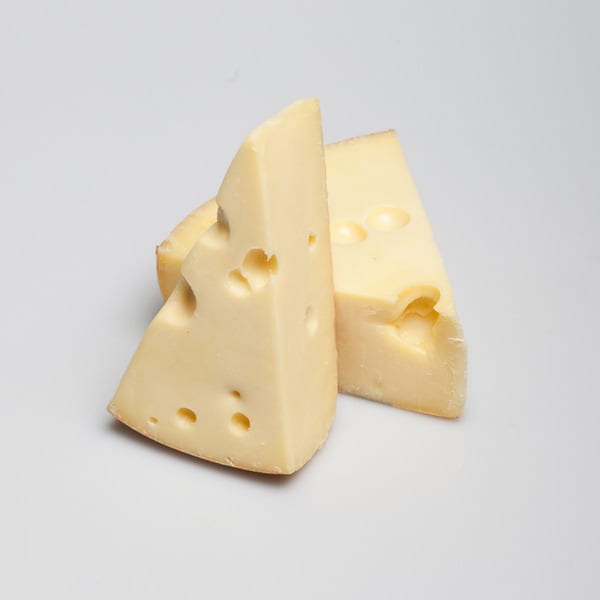** | **Cows**  **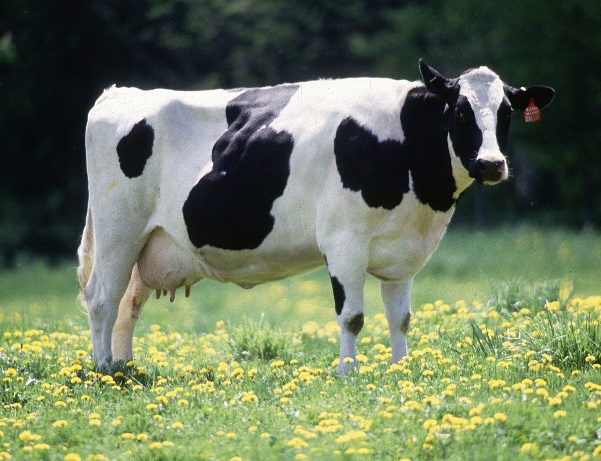** |
| 1. **Bacon**   **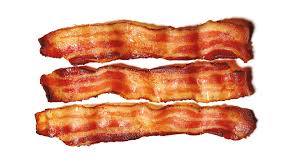** | **Bees**  **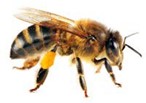** |

**Supply Chain**

1. **Harriet is a Year 4 student who lives in the city and likes to eat eggs for breakfast. What are the steps an egg goes through to get Harriet’s breakfast plate?**

**Draw a line to match up steps 1 – 5 to the picture that describes each step. Remember, the picture that comes first is step 1.**

| **STEP 1** | The carton of eggs travels to the supermarket or shop. | **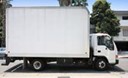** |
| --- | --- | --- |
|  |  | |
| **STEP 2** | Harriet or a family member purchases the eggs. | **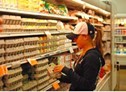** |
|  |  | |
| **STEP 3** | A hen lays an egg at a farm. | **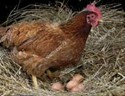** |
|  |  | |
| **STEP 4** | Harriet or someone in her family cooks the egg to eat. | **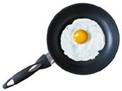** |
|  |  | |
| **STEP 5** | The eggs are washed and packaged in a carton. | **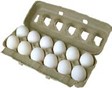** |

**Finding Nutrition Information**

1. **A box of cereal has a lot of information and pictures on it. Where on the box below would you find the best source of information about how healthy the food is?**
2. On the front of the box
3. On the side of the box
4. I don’t know

| **Front of Box** |  | **Side of box** |
| --- | --- | --- |
| 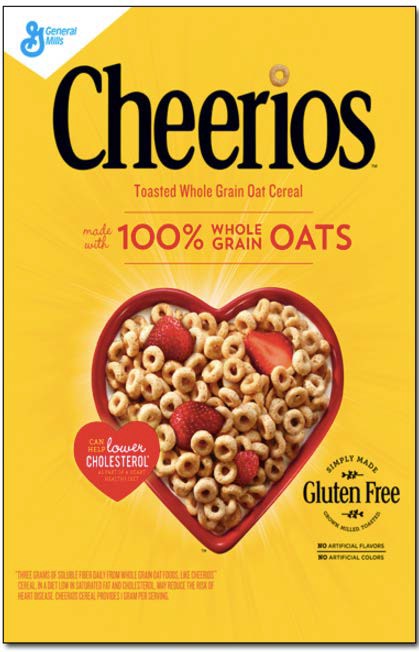 |  | 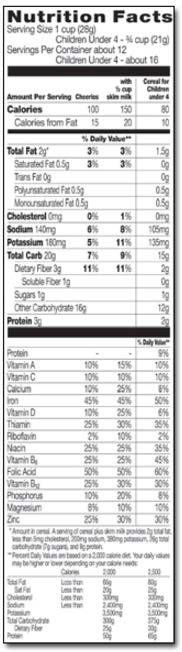 |

1. **It is recommended that you eat foods that are low in sugar. Based on the Nutrition Fact labels of two different cereal packets shown below, circle which one is lower in sugar.**
2. Cereal packet A
3. Cereal packet B
4. I don’t know

| **Cereal A** | **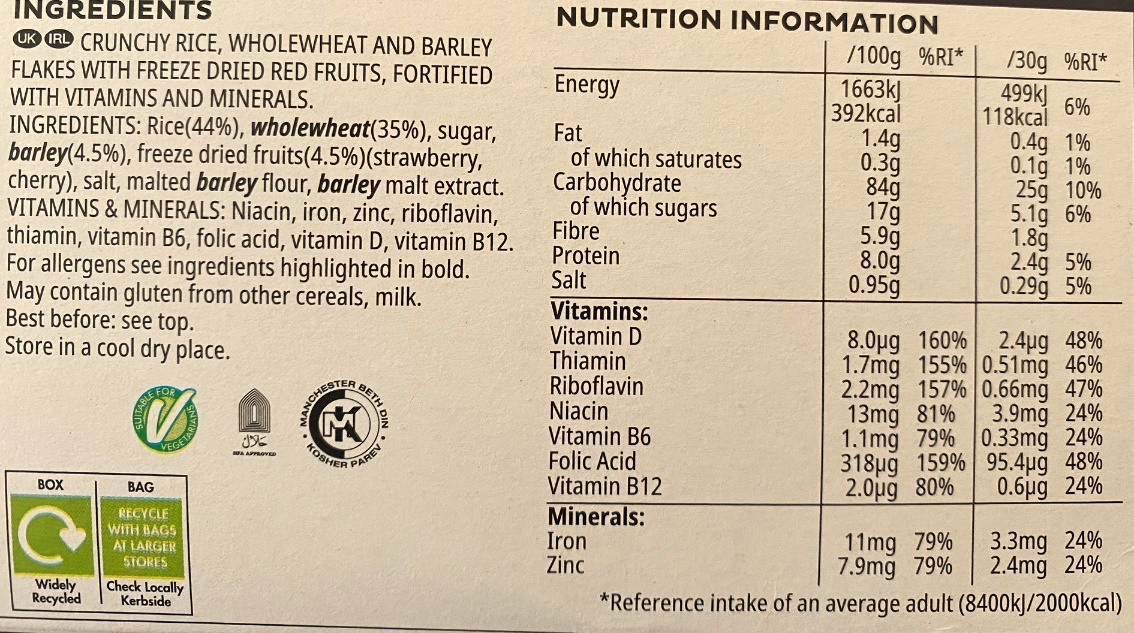** |
| --- | --- |
| **Cereal B** | **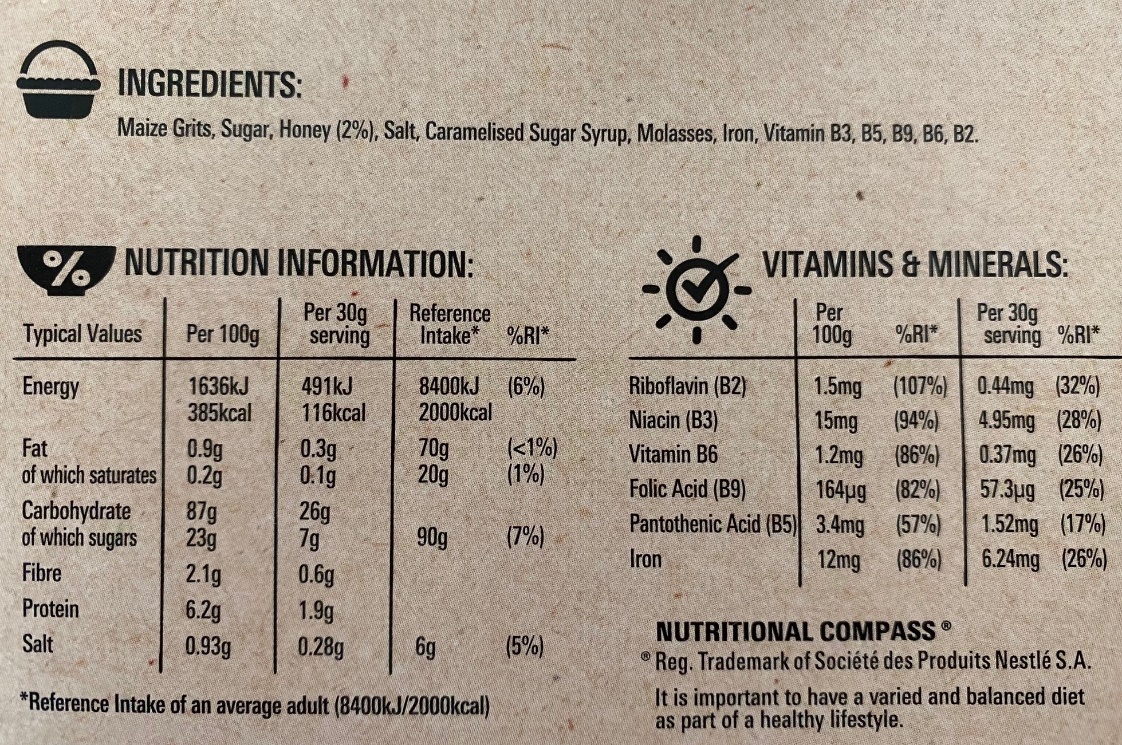** |

**Food and Your Body**

1. **Circle the best answer. The energy you get from eating healthy foods helps you to _______________.**
2. Move and play
3. Build muscles and bone
4. Keep your heart pumping blood
5. All of the above
6. I don’t know

**Food Groups**

**Questions #24-29: To stay healthy, it is important that we eat foods from five different food groups. Please draw a line to match each food below with their food group.**

| 1. Banana | Does not fit in a food group |
| --- | --- |
| 1. Grilled chicken | Vegetable |
| 1. Green beans | Dairy |
| 1. Brown bread | Carbohydrate |
| 1. Cheese | Protein |
| 1. Fruit-flavoured sweets | Fruit |

1. **A healthy meal should have a variety of foods from the five food groups. One of the best ways to plan a healthy meal is to be sure which of the food groups makes up 1/3 (a third) of your plate?**

**
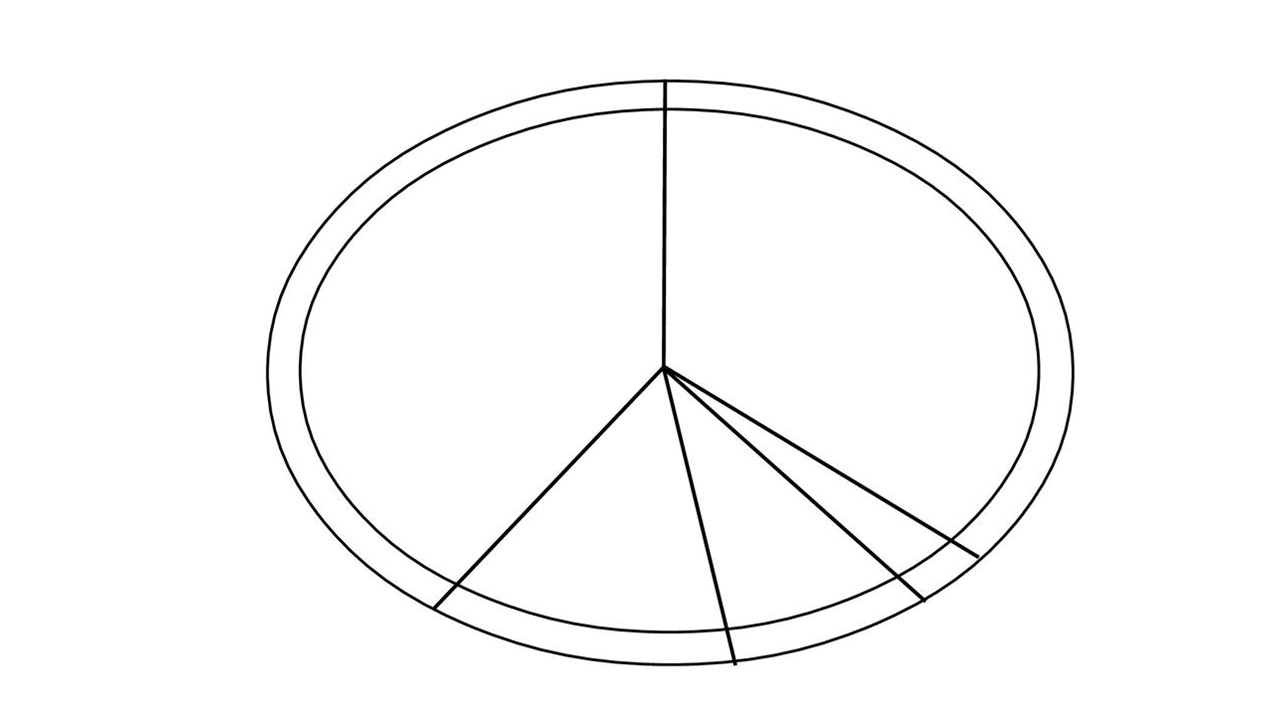
**

1. Dairy
2. Carbohydrate
3. Protein
4. Fruit and vegetables
5. I don’t know

**Eating with friends and family**

**Questions #31-34: Circle the letter to choose whether you think that you could do the following things to eat more fruit and vegetables.**

1. **For meals and snacks at home I can try a vegetable that an adult serves me.**
2. I think I can do this.
3. I am not sure I can do this.
4. I don’t think I can do this.
5. **For meals and snacks at home I can try a fruit that an adult serves me.**
6. I think I can do this.
7. I am not sure I can do this.
8. I don’t think I can do this.
9. **When eating lunch with a friend I can eat fruit even if my friend is not.**
10. I think I can do this.
11. I am not sure I can do this.
12. I don’t think I can do this.
13. **When eating lunch with a friend I can eat vegetables even if my friend says they are “yucky”.**
14. I think I can do this.
15. I am not sure I can do this.
16. I don’t think I can do this.

**STOP! You have finished taking the survey. Thank you! Please turn this survey over with the front page facing up and wait until an adult gives more instructions.**

1. Amin SA, Lehnerd M, Cash SB, Economos CD, Sacheck JM. Development of a Tool for Food Literacy Assessment in Children (TFLAC). J Nutr Educ Behav. 2019 Mar;51(3):364-369. doi: 10.1016/j.jneb.2018.12.006. PMID: 30851841. [↑](#footnote-ref-1)
