# Supplementary material for: Evaluation of the school-based ‘PhunkyFoods’ intervention: a cluster randomised controlled trial in the UK
Source: Public Health Nutr. 2025 Apr 14;28(1):e86. doi: 10.1017/S1368980025000552 (PMC12100561; doi:10.1017/S1368980025000552)
Supplement: Vaughan et al. supplementary material 2 — Vaughan et al. supplementary material [file S1368980025000552sup002.docx]

| Phunky Foods programme |  |  |  |  |  |  |  |  |  |
| --- | --- | --- | --- | --- | --- | --- | --- | --- | --- |
| CADET Fruit and Veg – Follow Up Survey March 2023 |  |  |  |  |  |  |  |  |  |
|  |  | | | | | | | | |

| **CADET: Child and Diet Evaluation Tool**  ***Fruit and Vegetables*** | **Home Diary** |
| --- | --- |

**This diary belongs to:**

| Name: |  |  |
| --- | --- | --- |
|  |  |  |
| Year Group: |  |  |
|  |  |  |
| School: |  |  |
|  |  |  |
| Today’s date: |  |  |

Dear Parent or Carer,

This diary will record the amount of fresh fruit and vegetables that your child eats for 24 hours - from 9am today to 9am tomorrow.

- All you need to do is to tick the fruit and vegetables your child eats at home and at school.
- If you child ate with someone else after school, ask your child or your child’s carer what they ate and tick if fruit and vegetables if they were consumed.

**HOW TO FILL IN THE CADET FRUIT AND VEGETABLES DIARY**

- Starting with the column headed “**Lunch (at school)**” ask your child what they ate at school and tick all the fruit and vegetables that they consumed for lunch that day.
- In the column headed **“Before tea (after school)”** tick all the fruit and vegetables that your child eats after finishing school today until their evening meal.
- In the column headed **“Evening meal/tea”**, tick all the fruit and vegetables your child had for their evening meal.
- In the column headed “**After tea/during night”** tick all the fruit and vegetables your child had after their evening meal and during the night.
- In the column headed “**Breakfast/before school”,** tick all the items of fruit and vegetables your child had at home before going to school the following morning.
- Make sure you ask your child if she/he ate any fruit or vegetables between leaving school and getting home. (if your child attended an after-school club, you should tick any fruit or vegetables your child consumed).

There are some additional questions that we would like you to complete at the end of the diary (page 6). When the diary is completed, please make sure it is placed in your child’s bag and sent back to school in the envelop provided.

Here are some examples of how to fill in CADET.

| 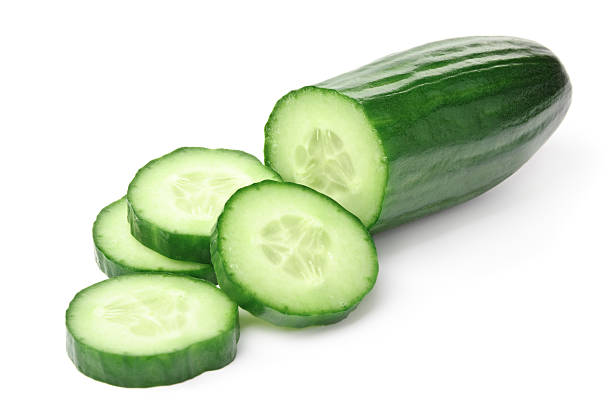 | Oscar had some cucumber on a sandwich at school so his mum ticked 🗹 this food in the column ‘**Lunch (at school)’** |
| --- | --- |

| **M** | **VEGETABLES & BEANS** | **Lunch (at school)** | | | **Before tea (after school)** | | | **Evening meal / tea** | | | **After tea / during night** | | | **Breakfast / before school** | | |
| --- | --- | --- | --- | --- | --- | --- | --- | --- | --- | --- | --- | --- | --- | --- | --- | --- |
|  |  |  |  |  |  |  |  |  |  |  |  |  |  |  |  |  |
| **1** | Cucumber |  | ✓ |  |  |  |  |  |  |  |  |  |  |  |  |  |
|  |  |  |  |  |  |  |  |  |  |  |  |  |  |  |  |  |
| **2** | Tomatoes |  |  |  |  |  |  |  |  |  |  |  |  |  |  |  |

|  | Oscar had a banana when he got home from school so his mum ticked 🗹 the column ‘**Before tea (after school)’** |
| --- | --- |

| **N** | **FRUIT** | **Lunch (at school)** | | | **Before tea (after school)** | | | **Evening meal / tea** | | | **After tea / during night** | | | **Breakfast / before school** | | |
| --- | --- | --- | --- | --- | --- | --- | --- | --- | --- | --- | --- | --- | --- | --- | --- | --- |
|  |  |  |  |  |  |  |  |  |  |  |  |  |  |  |  |  |
| **1** | Apple |  |  |  |  |  |  |  |  |  |  |  |  |  |  |  |
|  |  |  |  |  |  |  |  |  |  |  |  |  |  |  |  |  |
| **2** | Pear |  |  |  |  |  |  |  |  |  |  |  |  |  |  |  |
|  |  |  |  |  |  |  |  |  |  |  |  |  |  |  |  |  |
| **3** | Banana |  |  |  |  | ✓ |  |  |  |  |  |  |  |  |  |  |

Now complete the diary on pages 4 – 5 and questions on page 6

| **N** | **FRUIT** | **Lunch (at school)** | | | **Before tea (after school)** | | | **Evening meal / tea** | | | **After tea / during night** | | | **Breakfast / before school** | | |
| --- | --- | --- | --- | --- | --- | --- | --- | --- | --- | --- | --- | --- | --- | --- | --- | --- |
|  |  |  |  |  |  |  |  |  |  |  |  |  |  |  |  |  |
| **1** | Apple |  |  |  |  |  |  |  |  |  |  |  |  |  |  |  |
|  |  |  |  |  |  |  |  |  |  |  |  |  |  |  |  |  |
| **2** | Pear |  |  |  |  |  |  |  |  |  |  |  |  |  |  |  |
|  |  |  |  |  |  |  |  |  |  |  |  |  |  |  |  |  |
| **3** | Banana |  |  |  |  |  |  |  |  |  |  |  |  |  |  |  |
|  |  |  |  |  |  |  |  |  |  |  |  |  |  |  |  |  |
| **4** | Orange, satsuma etc. |  |  |  |  |  |  |  |  |  |  |  |  |  |  |  |
|  |  |  |  |  |  |  |  |  |  |  |  |  |  |  |  |  |
| **5** | Grapes |  |  |  |  |  |  |  |  |  |  |  |  |  |  |  |
|  |  |  |  |  |  |  |  |  |  |  |  |  |  |  |  |  |
| **6** | Melon |  |  |  |  |  |  |  |  |  |  |  |  |  |  |  |
|  |  |  |  |  |  |  |  |  |  |  |  |  |  |  |  |  |
| **7** | Pineapple |  |  |  |  |  |  |  |  |  |  |  |  |  |  |  |
|  |  |  |  |  |  |  |  |  |  |  |  |  |  |  |  |  |
| **8** | Strawberry, raspberry etc. |  |  |  |  |  |  |  |  |  |  |  |  |  |  |  |
|  |  |  |  |  |  |  |  |  |  |  |  |  |  |  |  |  |
| **9** | Peach, nectarine, plum, apricot, mango |  |  |  |  |  |  |  |  |  |  |  |  |  |  |  |
|  |  |  |  |  |  |  |  |  |  |  |  |  |  |  |  |  |
| **10** | Kiwi |  |  |  |  |  |  |  |  |  |  |  |  |  |  |  |
|  |  |  |  |  |  |  |  |  |  |  |  |  |  |  |  |  |
| **11** | Fruit salad (tinned or fresh) |  |  |  |  |  |  |  |  |  |  |  |  |  |  |  |
|  |  |  |  |  |  |  |  |  |  |  |  |  |  |  |  |  |
| **12** | Other fresh fruit |  |  |  |  |  |  |  |  |  |  |  |  |  |  |  |
|  |  |  |  |  |  |  |  |  |  |  |  |  |  |  |  |  |
| **13** | Dried fruit |  |  |  |  |  |  |  |  |  |  |  |  |  |  |  |

| **M** | **VEGETABLES & PULSES** | **Lunch (at school)** | | | **Before tea (after school)** | | | **Evening meal / tea** | | | **After tea / during night** | | | **Breakfast / before school** | | |
| --- | --- | --- | --- | --- | --- | --- | --- | --- | --- | --- | --- | --- | --- | --- | --- | --- |
|  |  |  |  |  |  |  |  |  |  |  |  |  |  |  |  |  |
| **1** | Cucumber |  |  |  |  |  |  |  |  |  |  |  |  |  |  |  |
|  |  |  |  |  |  |  |  |  |  |  |  |  |  |  |  |  |
| **2** | Tomatoes |  |  |  |  |  |  |  |  |  |  |  |  |  |  |  |
|  |  |  |  |  |  |  |  |  |  |  |  |  |  |  |  |  |
| **3** | Celery |  |  |  |  |  |  |  |  |  |  |  |  |  |  |  |
|  |  |  |  |  |  |  |  |  |  |  |  |  |  |  |  |  |
| **4** | Coleslaw |  |  |  |  |  |  |  |  |  |  |  |  |  |  |  |
|  |  |  |  |  |  |  |  |  |  |  |  |  |  |  |  |  |
| **5** | Other salad vegetables, e.g. lettuce | |  |  |  |  |  |  |  |  |  |  |  |  |  |  |
|  |  |  |  |  |  |  |  |  |  |  |  |  |  |  |  |  |
| **6** | Stir-fried vegetables |  |  |  |  |  |  |  |  |  |  |  |  |  |  |  |
|  |  |  |  |  |  |  |  |  |  |  |  |  |  |  |  |  |
| **7** | Broccoli, brussel sprouts, cabbage |  |  |  |  |  |  |  |  |  |  |  |  |  |  |  |
|  |  |  |  |  |  |  |  |  |  |  |  |  |  |  |  |  |
| **8** | Carrots |  |  |  |  |  |  |  |  |  |  |  |  |  |  |  |
|  |  |  |  |  |  |  |  |  |  |  |  |  |  |  |  |  |
| **9** | Cauliflower |  |  |  |  |  |  |  |  |  |  |  |  |  |  |  |
|  |  |  |  |  |  |  |  |  |  |  |  |  |  |  |  |  |
| **10** | Peas, sweetcorn |  |  |  |  |  |  |  |  |  |  |  |  |  |  |  |
|  |  |  |  |  |  |  |  |  |  |  |  |  |  |  |  |  |
| **11** | Mixed vegetables |  |  |  |  |  |  |  |  |  |  |  |  |  |  |  |
|  |  |  |  |  |  |  |  |  |  |  |  |  |  |  |  |  |
| **12** | Celeriac / swede |  |  |  |  |  |  |  |  |  |  |  |  |  |  |  |
|  |  |  |  |  |  |  |  |  |  |  |  |  |  |  |  |  |
| **13** | Peppers, red, green, yellow etc. |  |  |  |  |  |  |  |  |  |  |  |  |  |  |  |
|  |  |  |  |  |  |  |  |  |  |  |  |  |  |  |  |  |
| **14** | Other vegetables |  |  |  |  |  |  |  |  |  |  |  |  |  |  |  |
|  |  |  |  |  |  |  |  |  |  |  |  |  |  |  |  |  |
| **15** | Baked beans |  |  |  |  |  |  |  |  |  |  |  |  |  |  |  |
|  |  |  |  |  |  |  |  |  |  |  |  |  |  |  |  |  |
| **16** | Lentils, Dahl |  |  |  |  |  |  |  |  |  |  |  |  |  |  |  |
|  |  |  |  |  |  |  |  |  |  |  |  |  |  |  |  |  |
| **17** | Other beans |  |  |  |  |  |  |  |  |  |  |  |  |  |  |  |
|  |  |  |  |  |  |  |  |  |  |  |  |  |  |  |  |  |
| **18** | Seeds, e.g. sunflower, sesame |  |  |  |  |  |  |  |  |  |  |  |  |  |  |  |
|  |  |  |  |  |  |  |  |  |  |  |  |  |  |  |  |  |

| **The following questions are about what you and your child think about eating fruit and vegetables.** Please tick the closest answer. | | | | | | | | | | | | | | | | |
| --- | --- | --- | --- | --- | --- | --- | --- | --- | --- | --- | --- | --- | --- | --- | --- | --- |
|  |  | **Yes always** | | | **Yes, most days / often** | | | **Sometimes** | | | **Seldom** | | | **Never** | | |
|  |  |  |  |  |  |  |  |  |  |  |  |  |  |  |  |  |
| **1** | We nearly always have different kinds of fruit at home. |  |  |  |  |  |  |  |  |  |  |  |  |  |  |  |
|  |  |  |  |  |  |  |  |  |  |  |  |  |  |  |  |  |
| **2** | How aften do you buy specific fruit / vegetables because your child asks for them? |  |  |  |  |  |  |  |  |  |  |  |  |  |  |  |
|  |  |  |  |  |  |  |  |  |  |  |  |  |  |  |  |  |
| **3** | How often do you cut up fruit and vegetables for your child to eat between meals? |  |  |  |  |  |  |  |  |  |  |  |  |  |  |  |
|  |  |  |  |  |  |  |  |  |  |  |  |  |  |  |  |  |
| **4** | I eat fruit / vegetables every day. |  |  |  |  |  |  |  |  |  |  |  |  |  |  |  |
|  |  |  |  |  |  |  |  |  |  |  |  |  |  |  |  |  |
| **5** | How often do you eat fruit / vegetables together with your child? |  |  |  |  |  |  |  |  |  |  |  |  |  |  |  |
|  |  |  |  |  |  |  |  |  |  |  |  |  |  |  |  |  |
| **6** | Do you have to persuade your child to eat fruit / vegetables? |  |  |  |  |  |  |  |  |  |  |  |  |  |  |  |
|  |  |  |  |  |  |  |  |  |  |  |  |  |  |  |  |  |
| **7** | How often do you have to ask your child to eat their fruit or vegetables? |  |  |  |  |  |  |  |  |  |  |  |  |  |  |  |
|  |  |  |  |  |  |  |  |  |  |  |  |  |  |  |  |  |

Thank you very much for completing the CADET fruit and vegetables diary. Please place the completed diary in the envelope provided and return to the school office.
