# Supplementary material for: Evaluation of the school-based ‘PhunkyFoods’ intervention: a cluster randomised controlled trial in the UK
Source: Public Health Nutr. 2025 Apr 14;28(1):e86. doi: 10.1017/S1368980025000552 (PMC12100561; doi:10.1017/S1368980025000552)
Supplement: Vaughan et al. supplementary material 4 — Vaughan et al. supplementary material [file S1368980025000552sup004.docx]

Key Stage 2 Child Survey – cooking skills

This survey is part of a research project with Phunky Foods and the University of Leeds. The questions are based on academic research on cooking skills^[[1]](#footnote-1)^. We want to find out about food literacy and cooking skills for children at Primary Schools. No individual names will be used as part of our research. You can ask a teacher to help you to complete this survey.

| Please put a tick in the box if you are happy for this survey to be used for research. ☺ |  |
| --- | --- |

**About you**

| Name of your school: |  | | |
| --- | --- | --- | --- |
|  |  | | |
| Your name: |  | | |
|  |  | | |
|  | Day | Month | Year |
| What is your date of birth? |  |  |  |
|  |  | | |
| What school year are you in now? |  | | |
|  |  |  |  |
| What is today’s date? | Day | Month | Year |
|  |  |  |  |

Question 1

**COOKING SKILLS SURVEY**


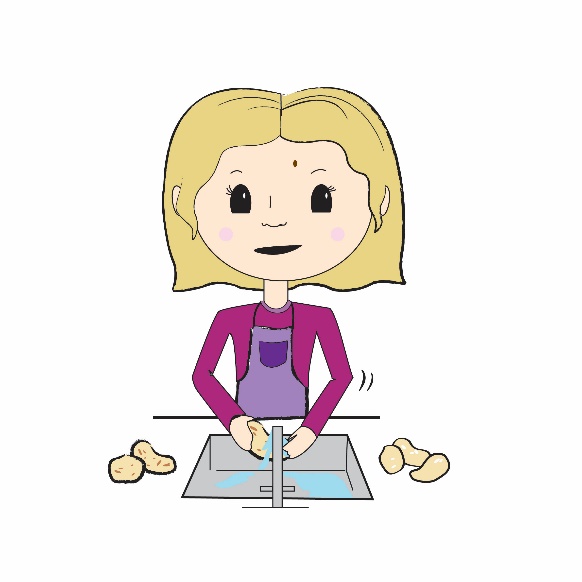


This child is washing vegetables. Do you do this?

□ Yes

□ No

Question 2

| 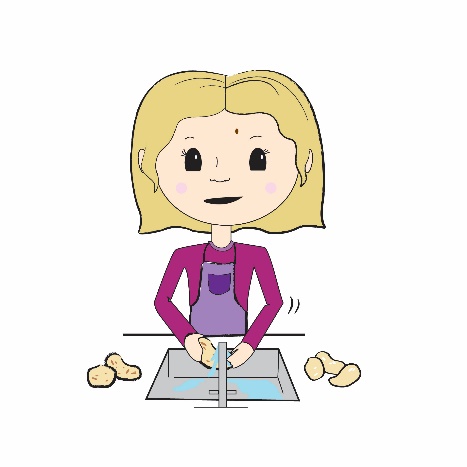 | 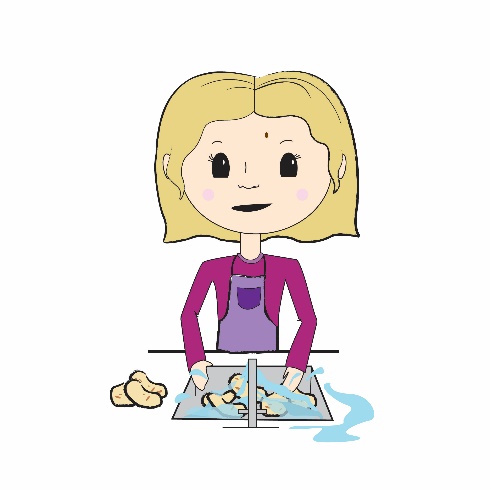 |
| --- | --- |
| A is really good at washing | B is not that good at washing |

Which are you MOST like?

□ I am a lot like A

□ I am a little like A

□ I am a bit like A and B

□ I am a little like B

□ I am a lot like BQuestion 3


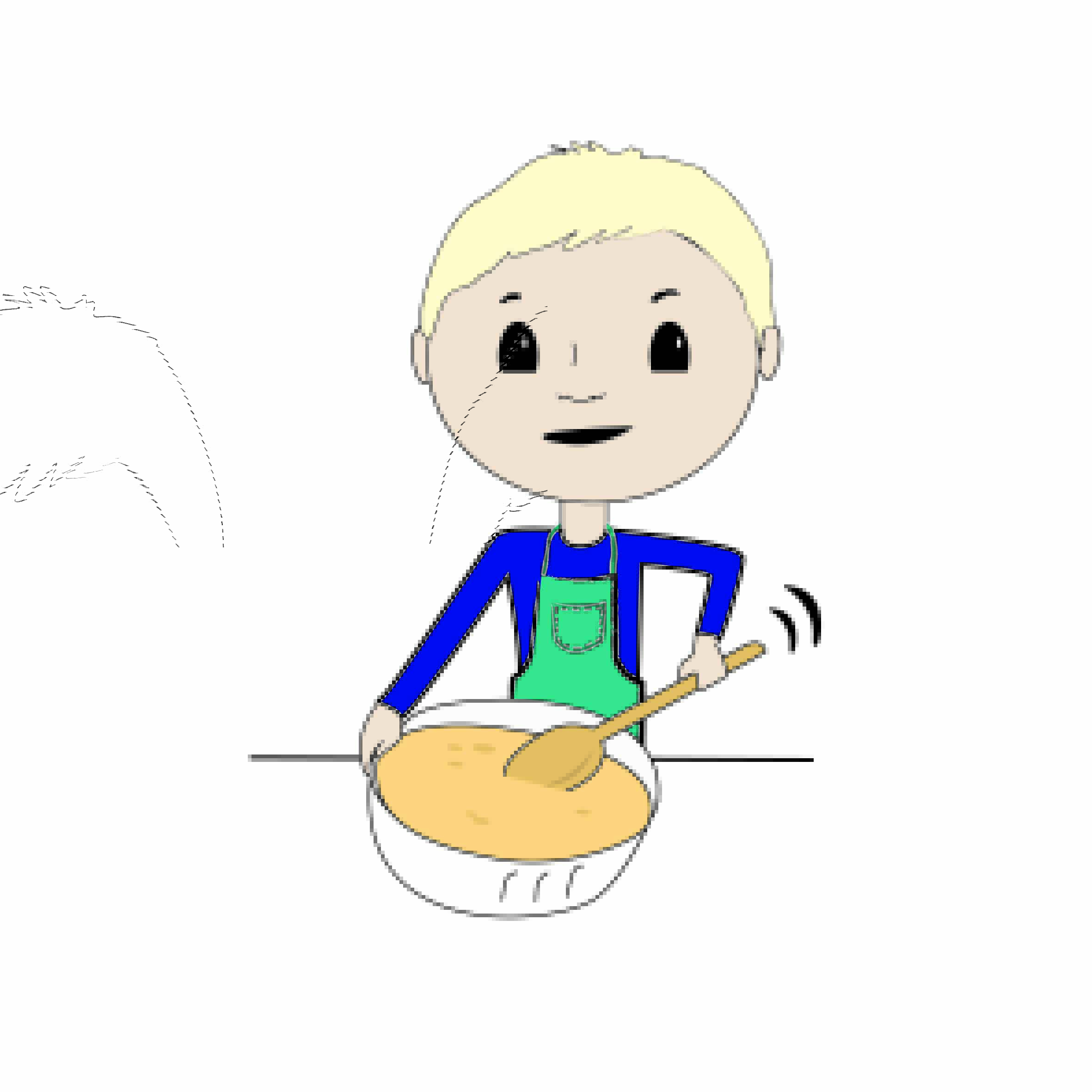


This child is stirring/mixing ingredients. Do you do this?

□ Yes

□ No

Question 4

| 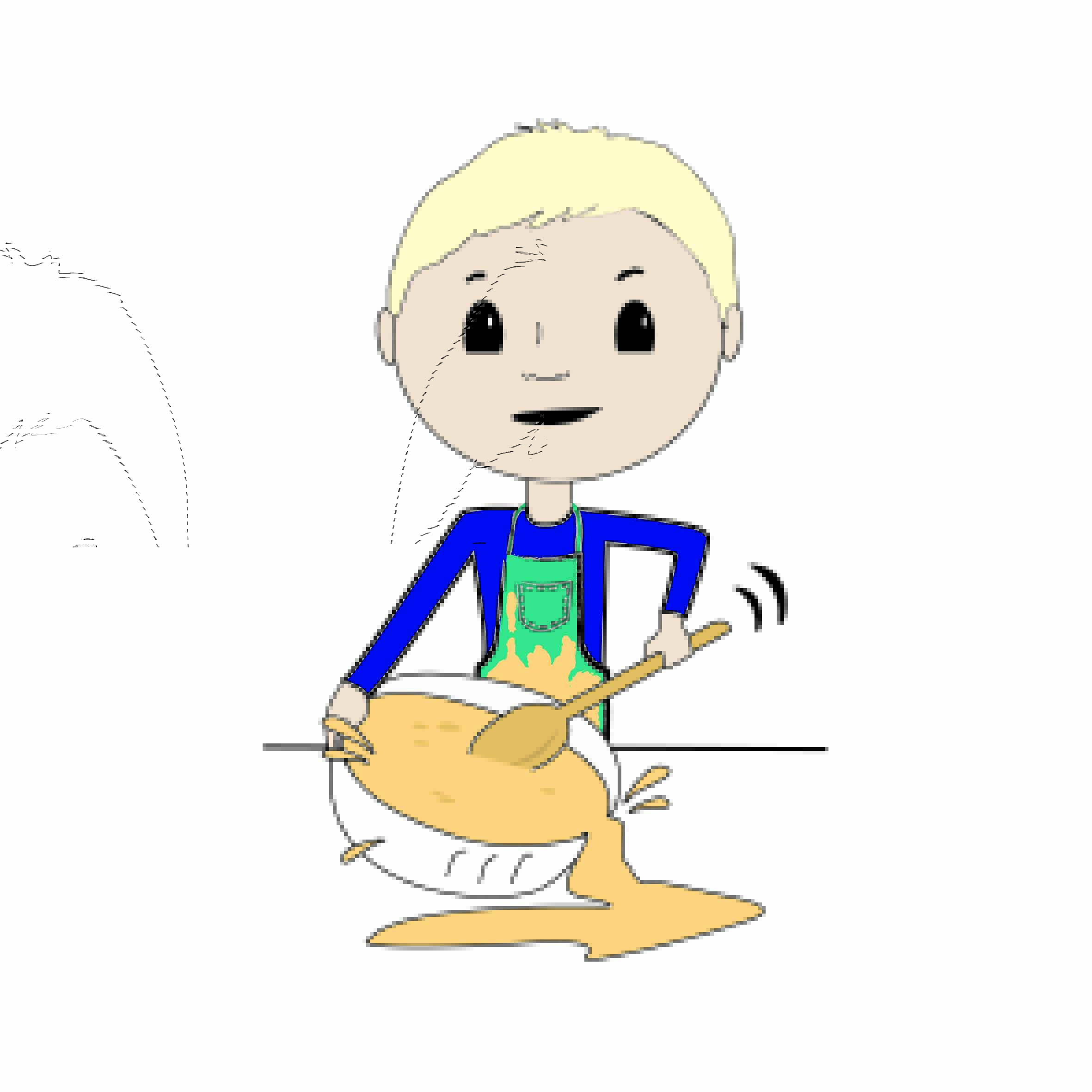 | 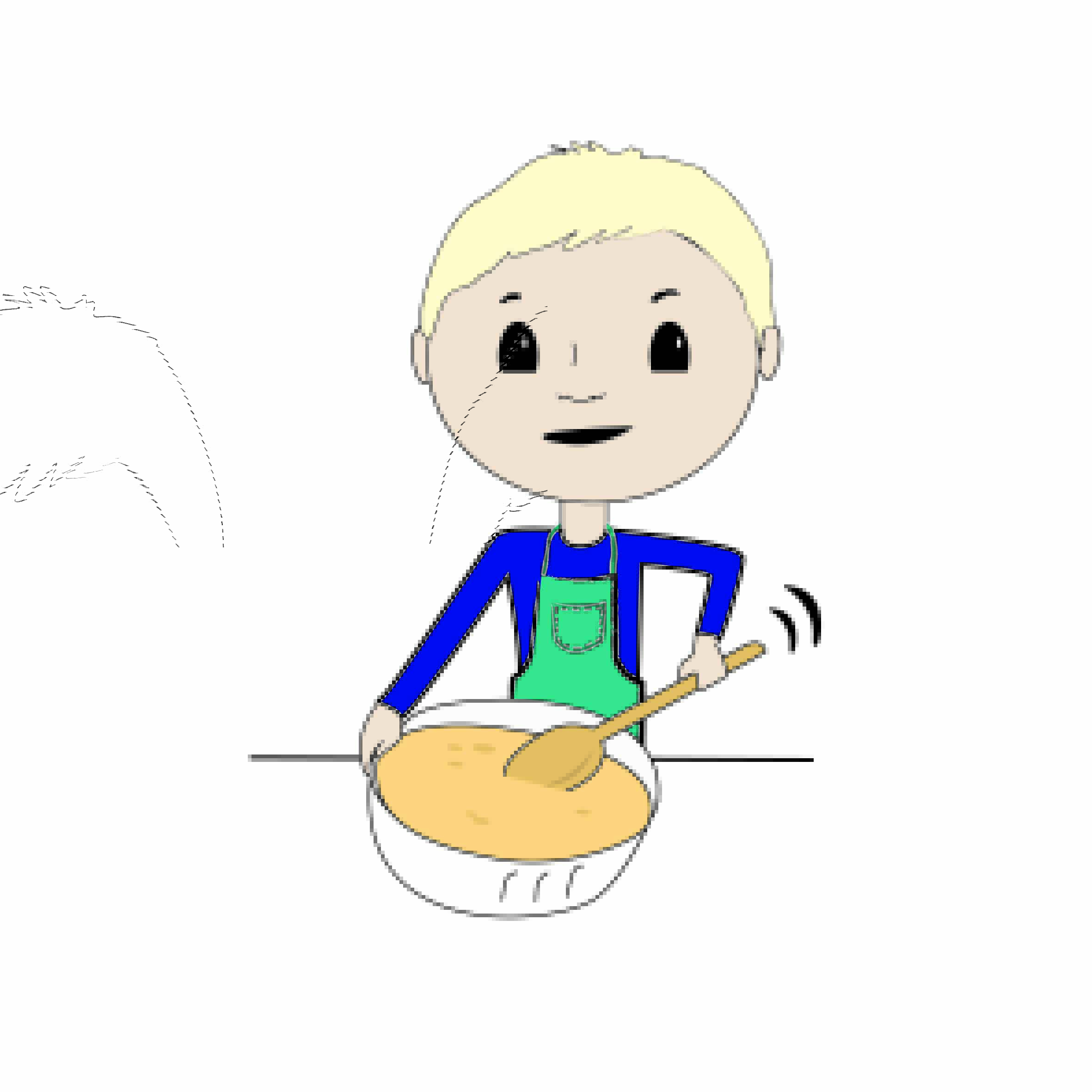 |
| --- | --- |
| A is not that good at stirring/mixing | B is really good at stirring/mixing |

Which are you MOST like?

□ I am a lot like A

□ I am a little like A

□ I am a bit like A and B

□ I am a little like B

□ I am a lot like B

Question 5


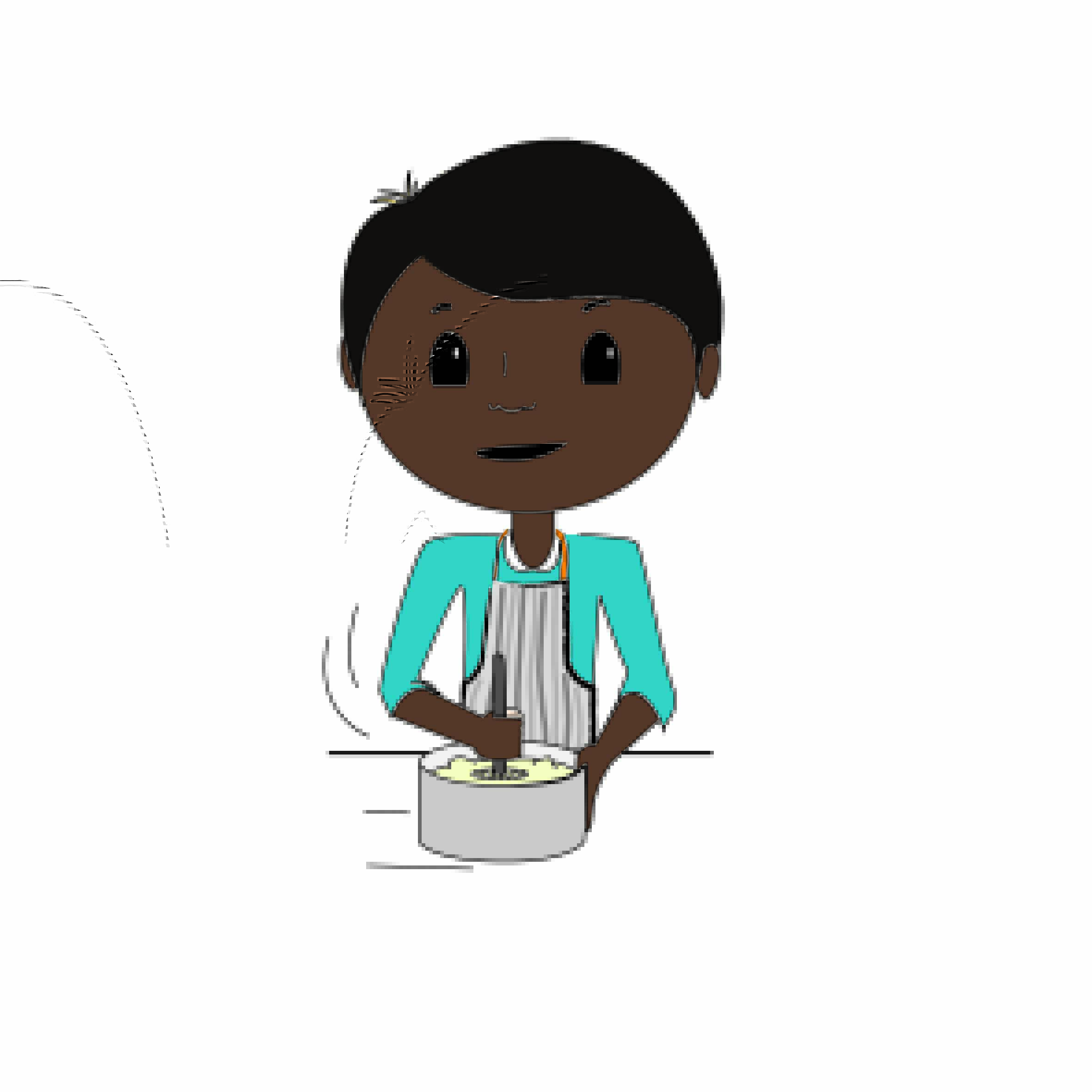


This child is mashing. Do you do this?

□ Yes

□ No

Question 6

| 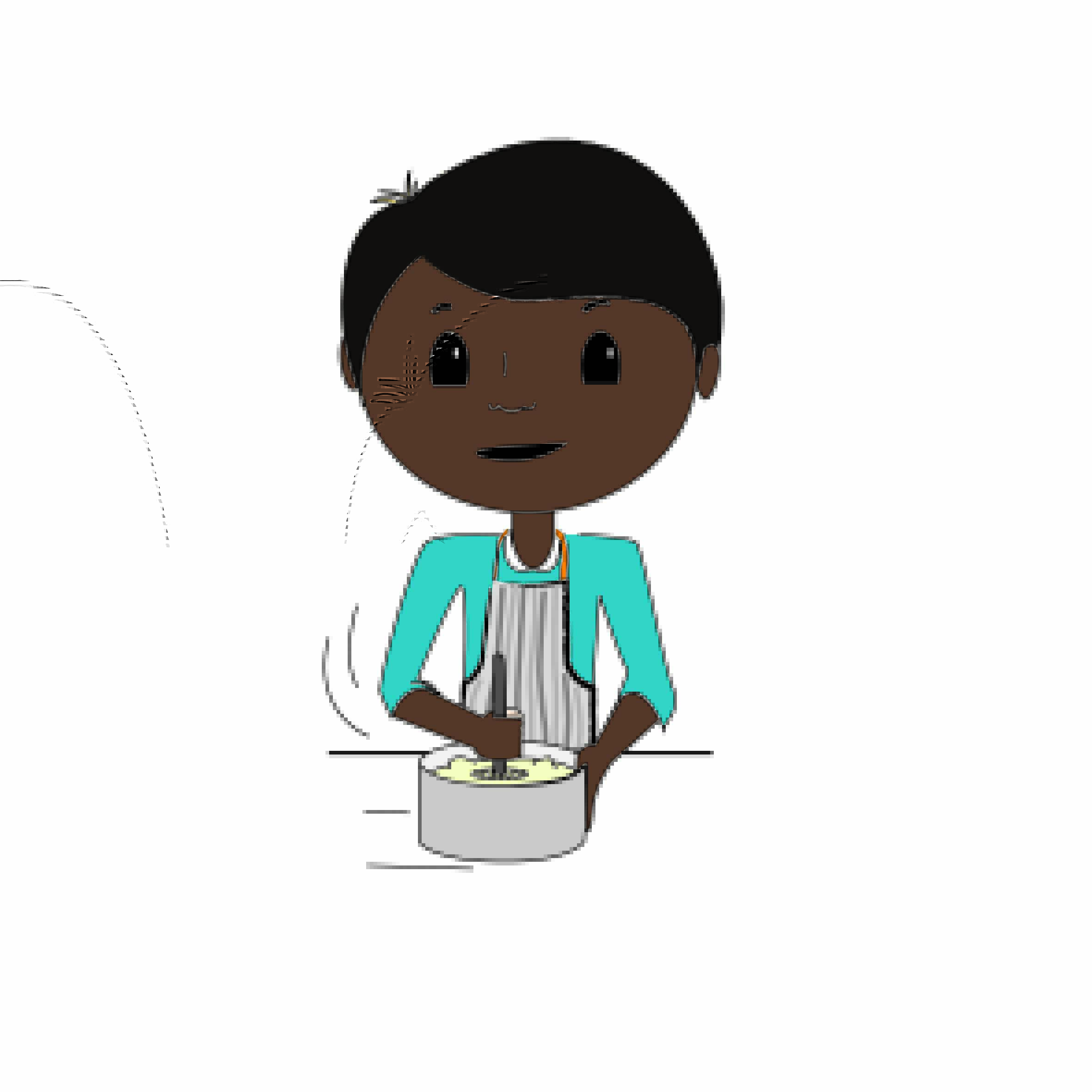 | 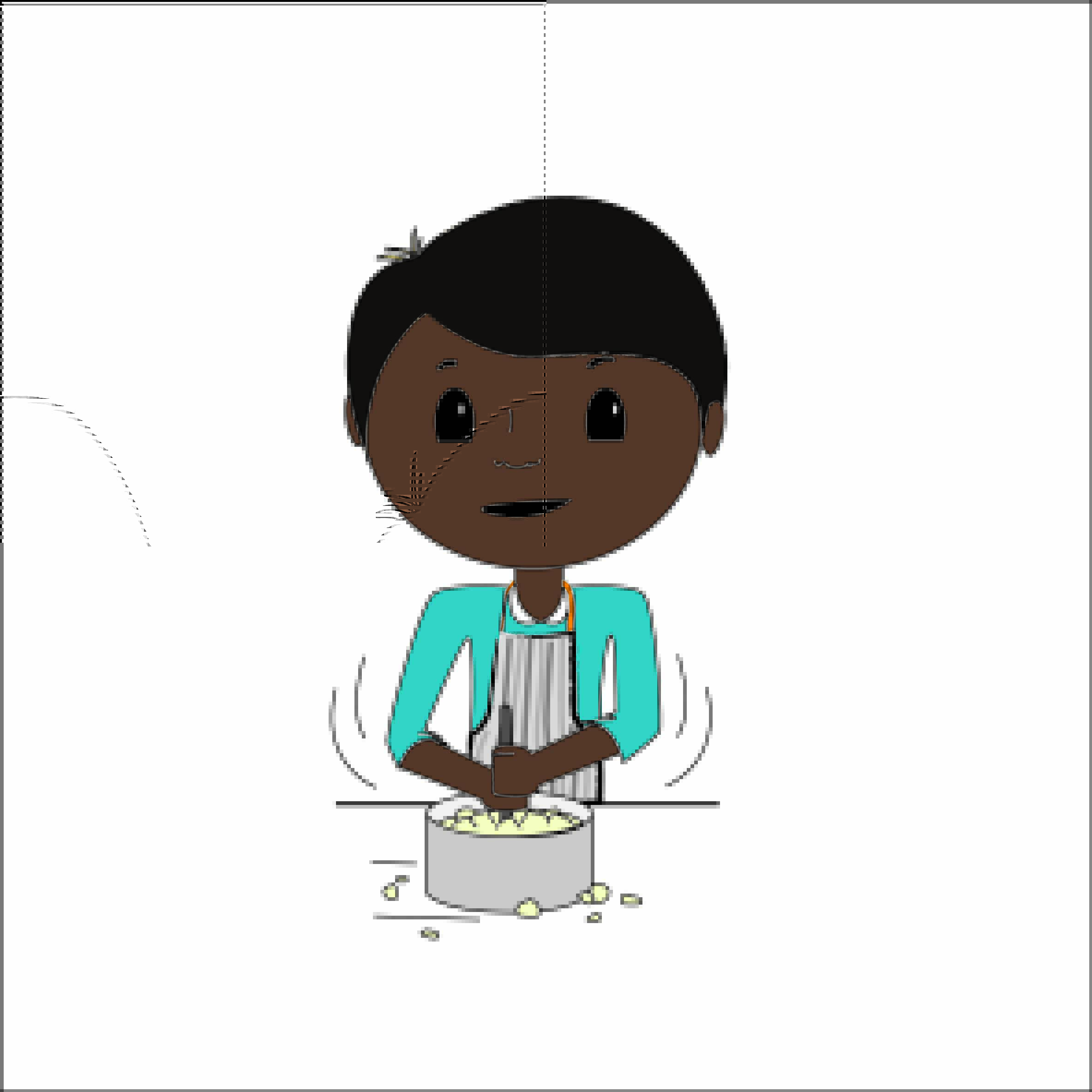 |
| --- | --- |
| A is really good at mashing | B is not that good at mashing |

Which are you MOST like?

□ I am a lot like A

□ I am a little like A

□ I am a bit like A and B

□ I am a little like B

□ I am a lot like B

Question 7


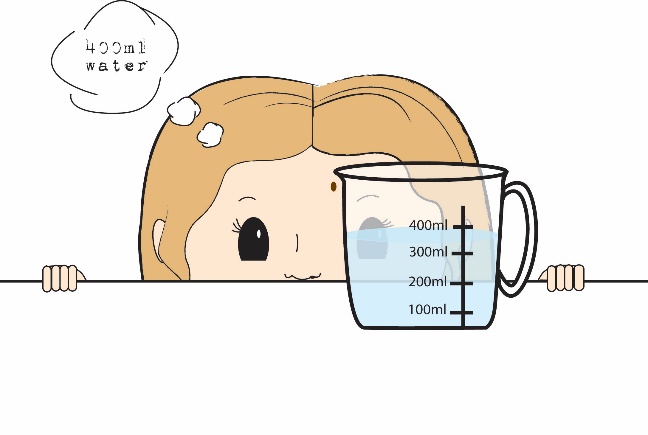


This child is measuring liquids. Do you do this?

□ Yes

□ No

Question 8

| 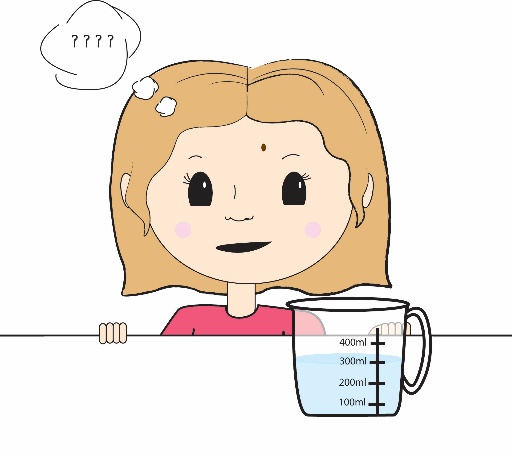 | 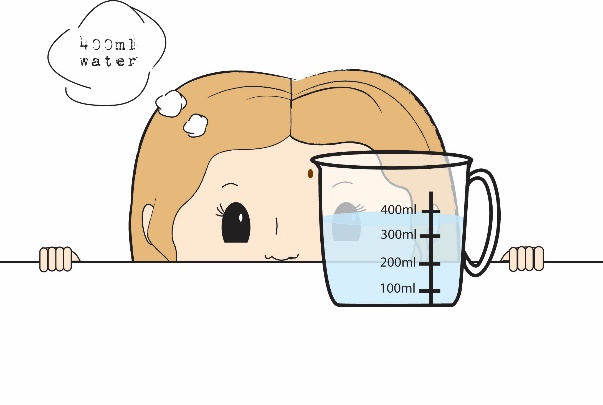 |
| --- | --- |
| A is not that good at measuring liquids | B is really good at measuring liquids |

Which are you MOST like?

□ I am a lot like A

□ I am a little like A

□ I am a bit like A and B

□ I am a little like B

□ I am a lot like B

Question 9


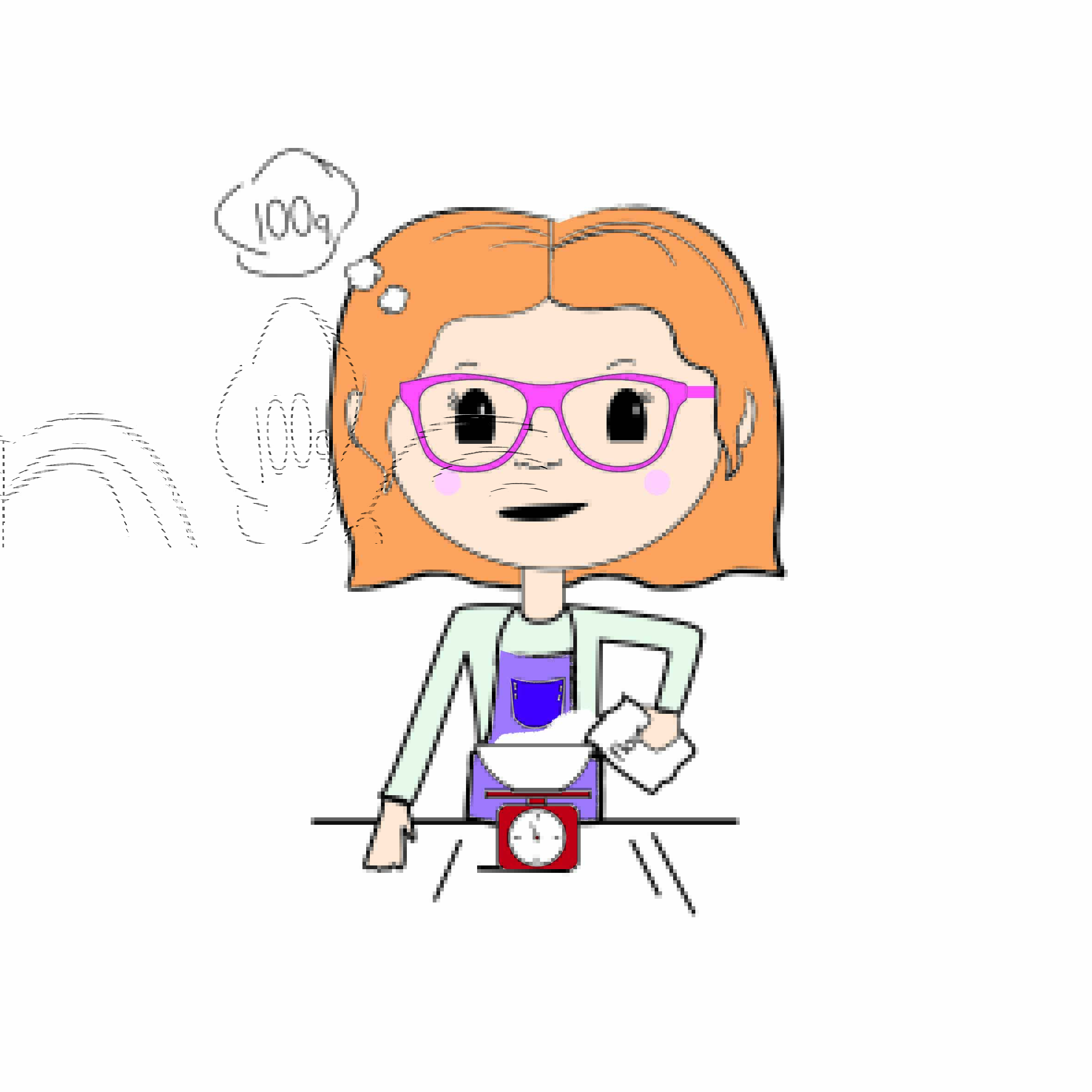


This child is weighing ingredients. Do you do this?

□ Yes

□ No

Question 10

| 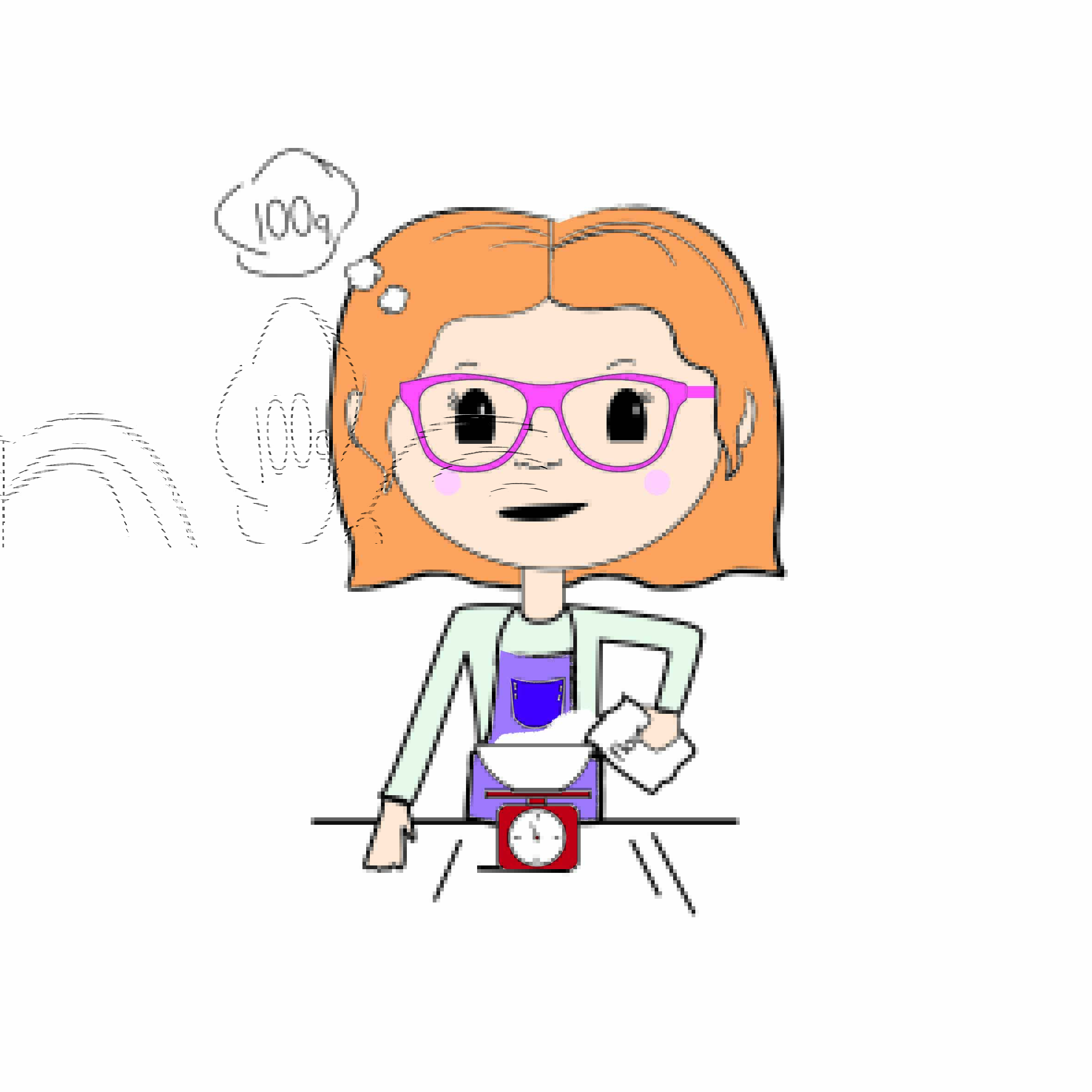 | 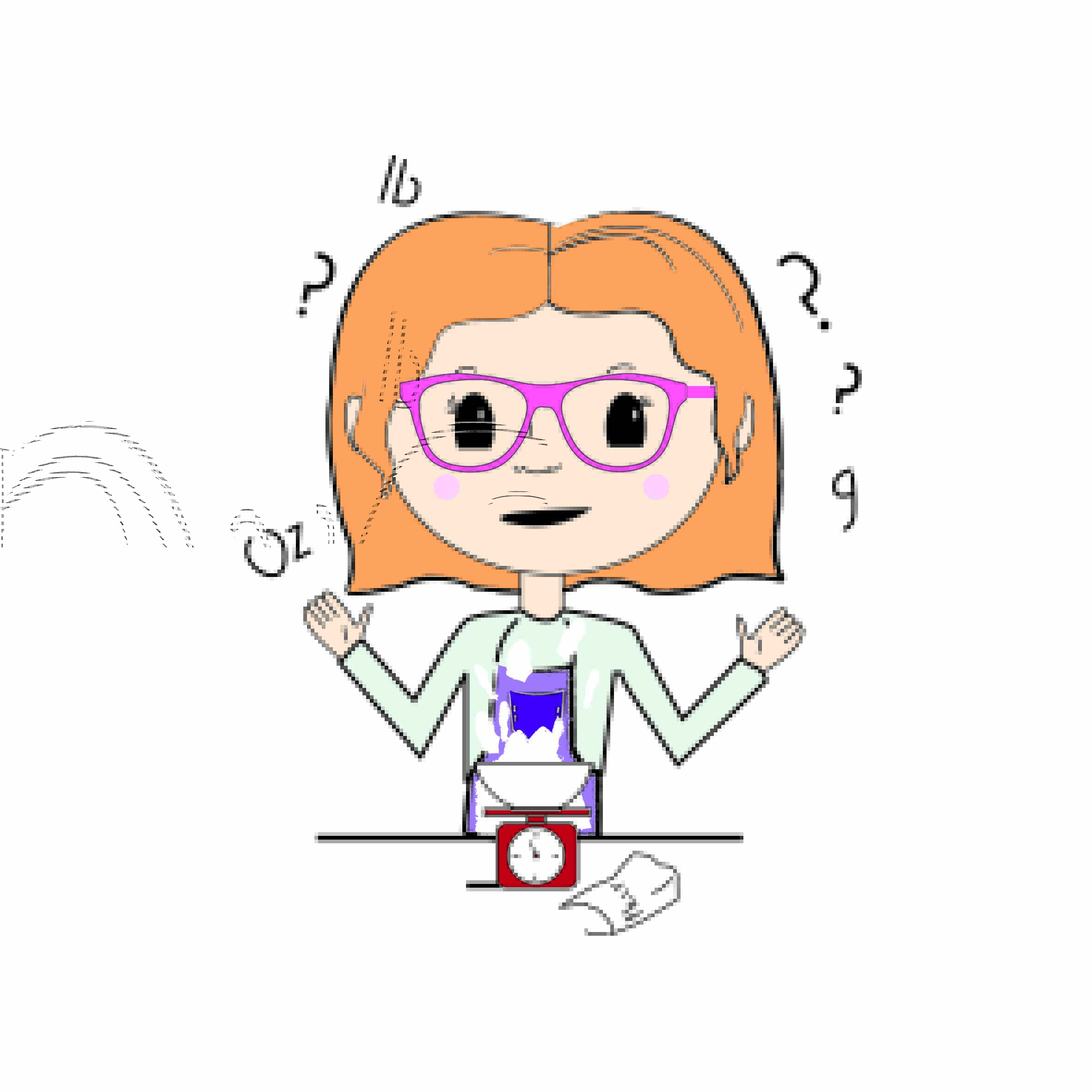 |
| --- | --- |
| A is really good at weighing | B is not that good at weighing |

Which are you MOST like?

□ I am a lot like A

□ I am a little like A

□ I am a bit like A and B

□ I am a little like B

□ I am a lot like B

Question 11


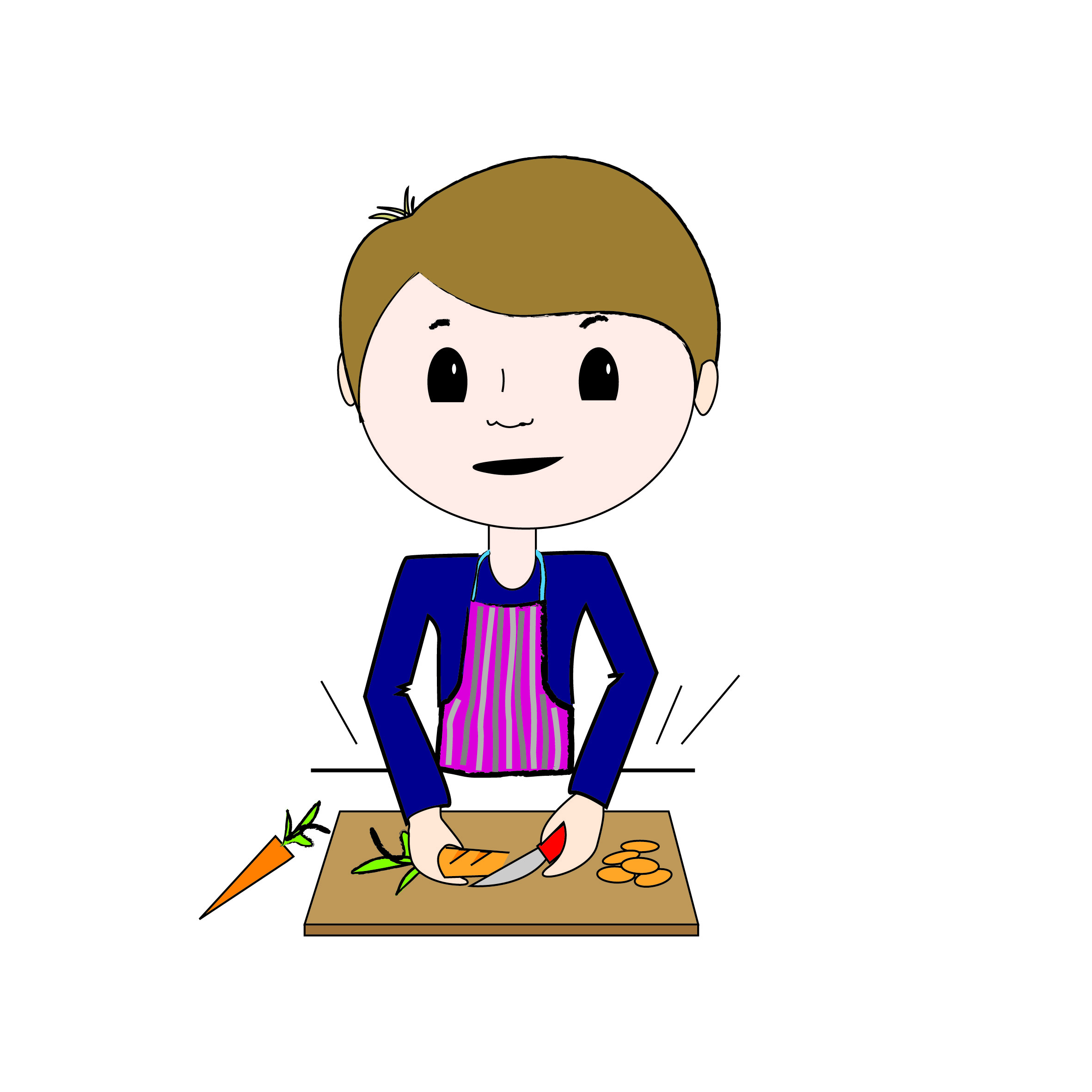


This child is chopping. Do you do this?

□ Yes

□ No

Question 12

| 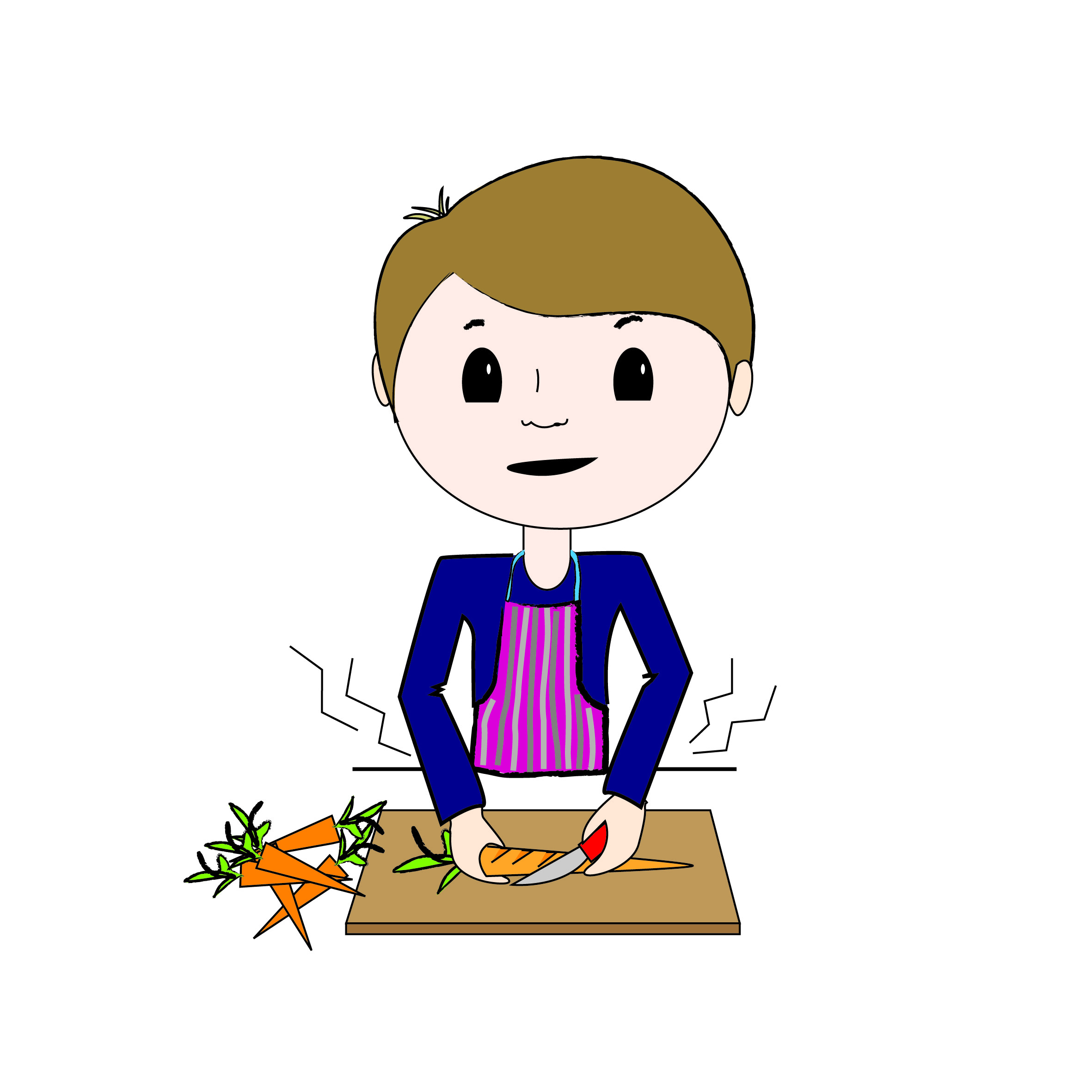 | 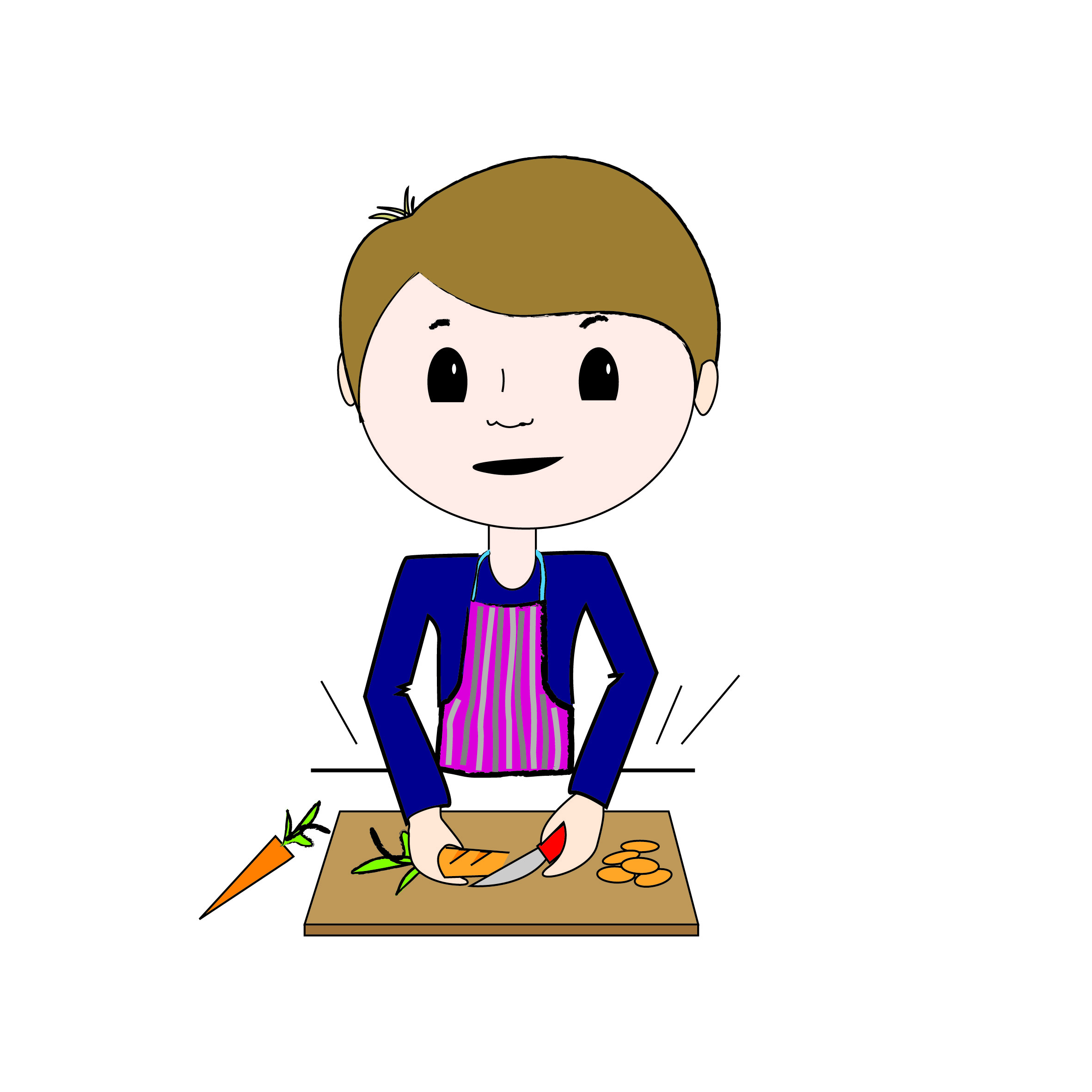 |
| --- | --- |
| A is not that good at chopping | B is really good at chopping |

Which are you MOST like?

□ I am a lot like A

□ I am a little like A

□ I am a bit like A and B

□ I am a little like B

□ I am a lot like B

Question 13


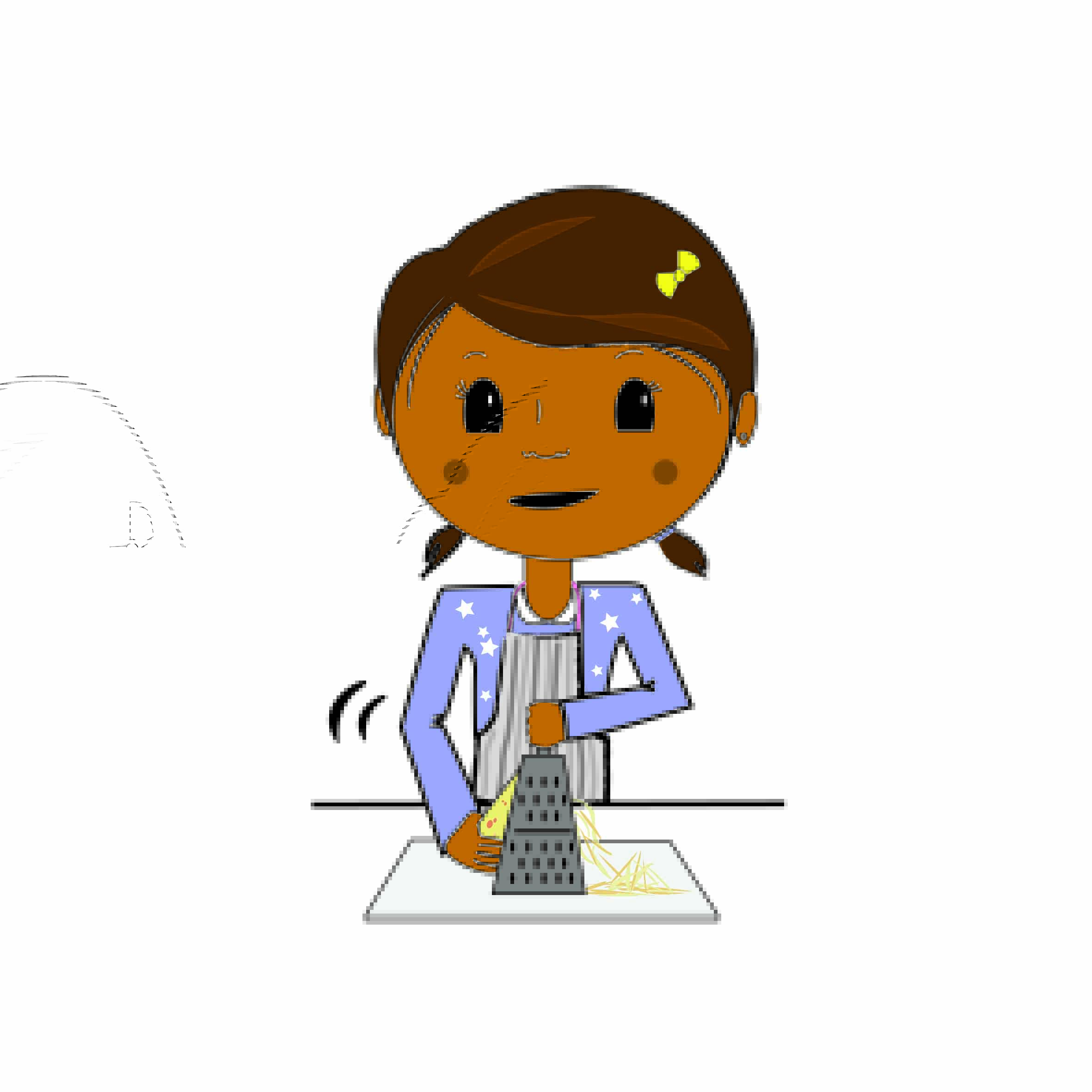


This child is grating. Do you do this?

□ Yes

□ No

Question 14

| 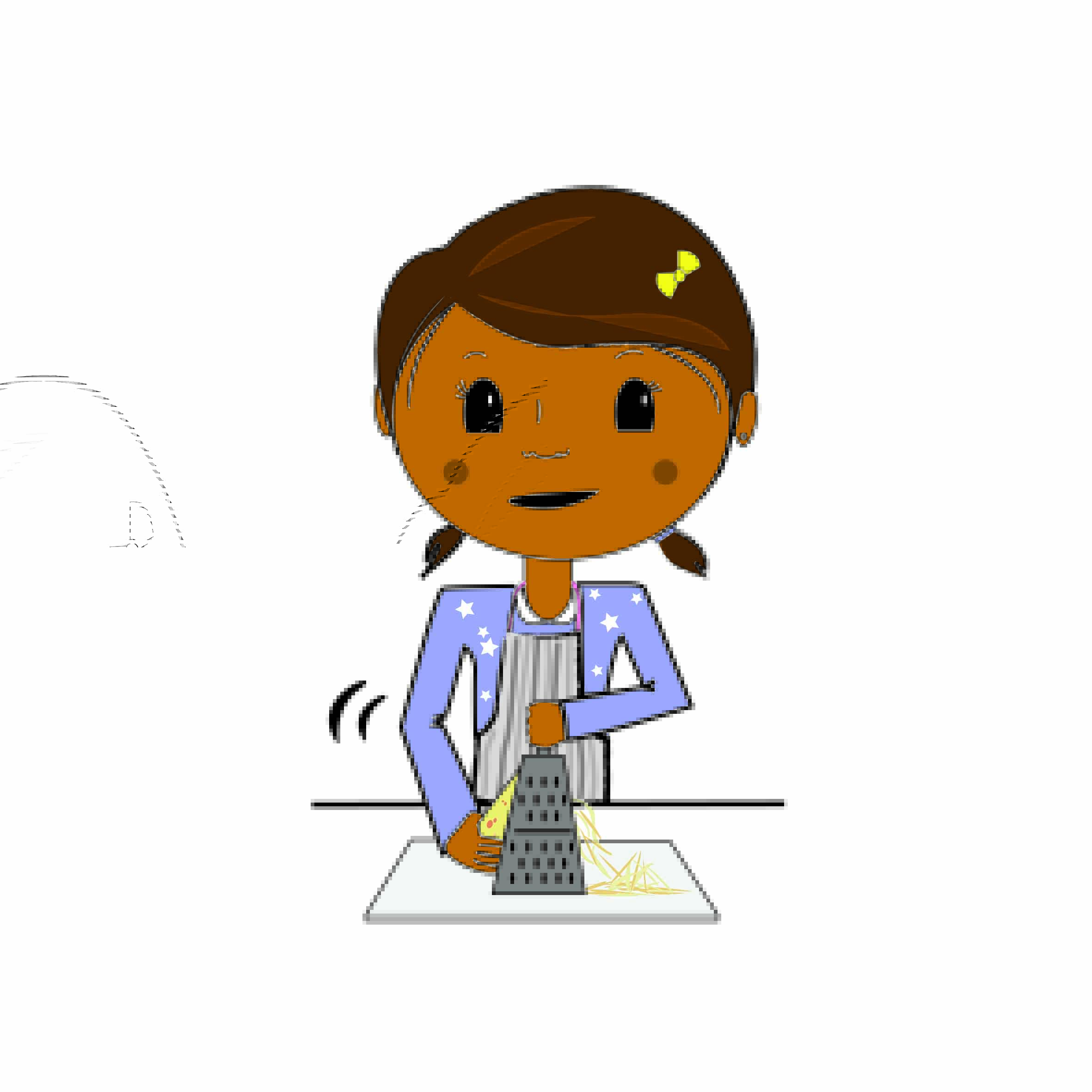 | 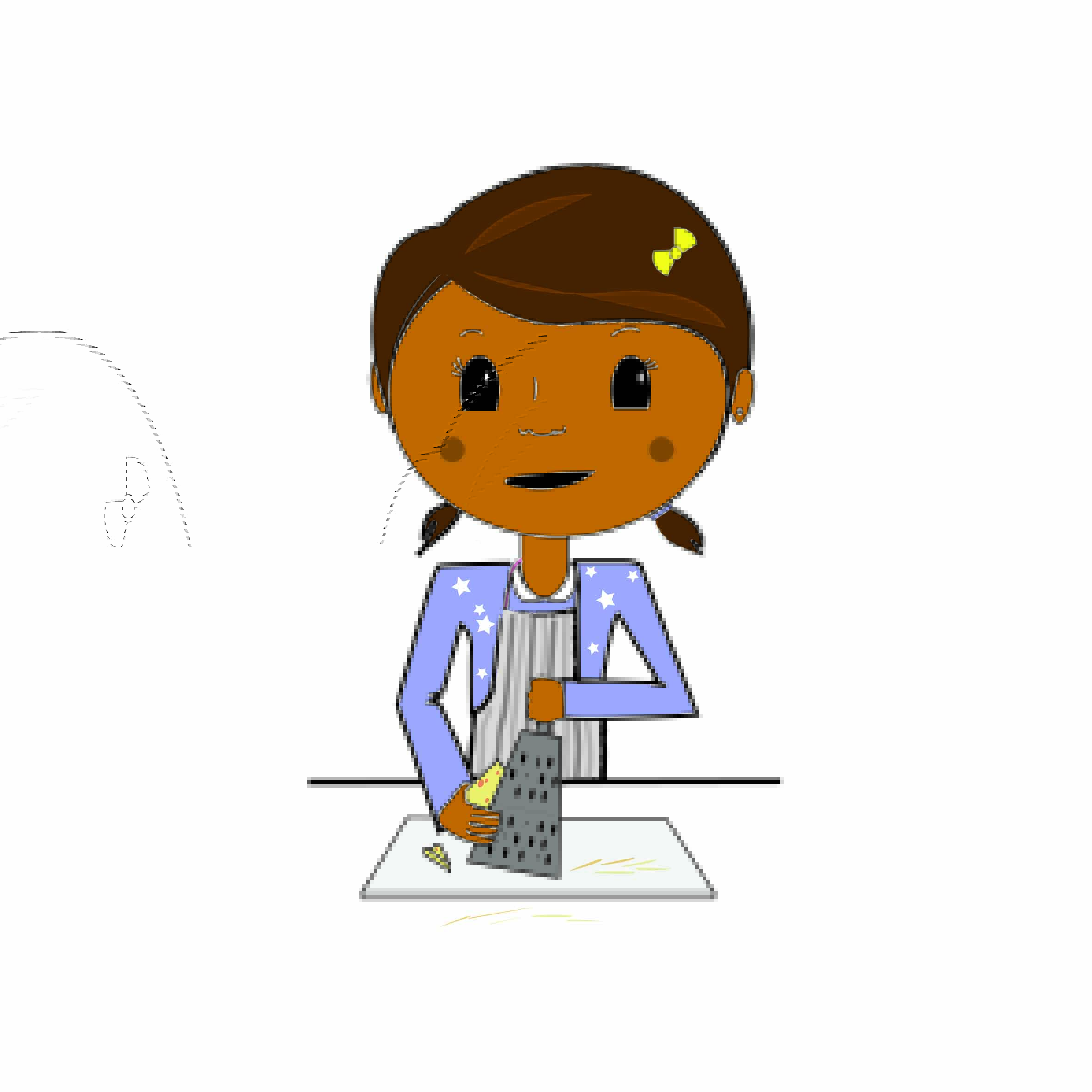 |
| --- | --- |
| A is really good at grating | B is not that good at grating |

Which are you MOST like?

□ I am a lot like A

□ I am a little like A

□ I am a bit like A and B

□ I am a little like B

□ I am a lot like B

Question 15


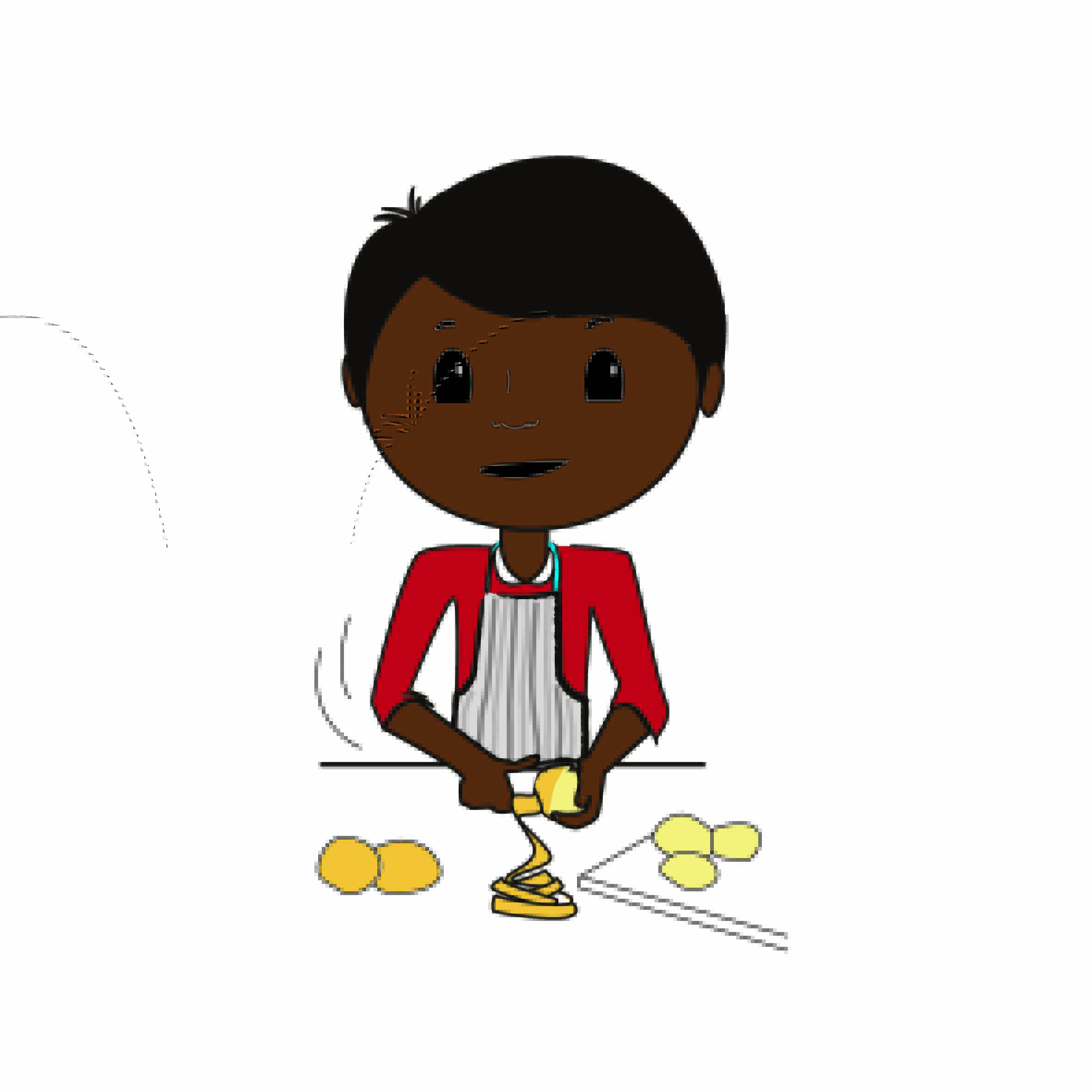


This child is peeling. Do you do this?

□ Yes

□ No

Question 16

| 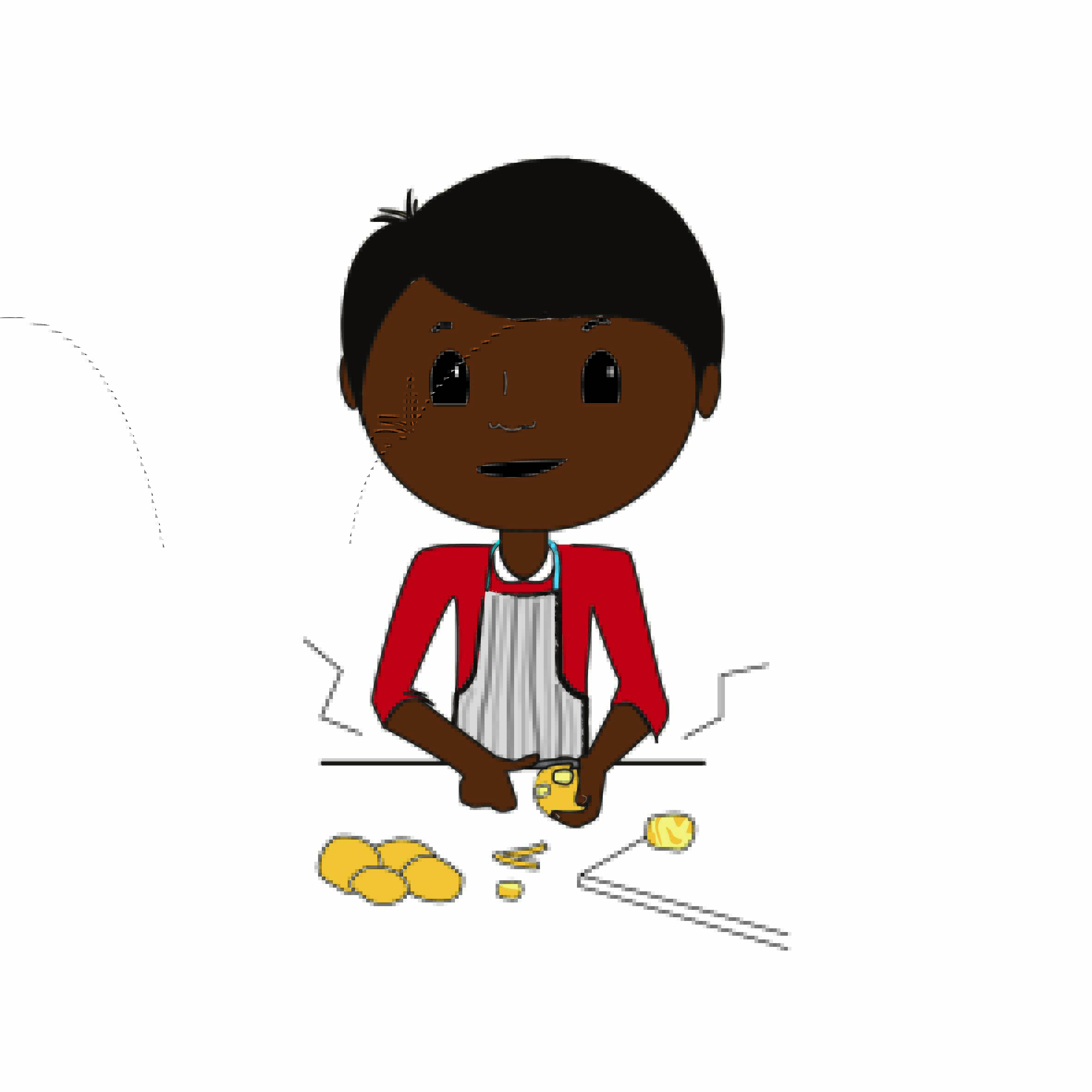 | 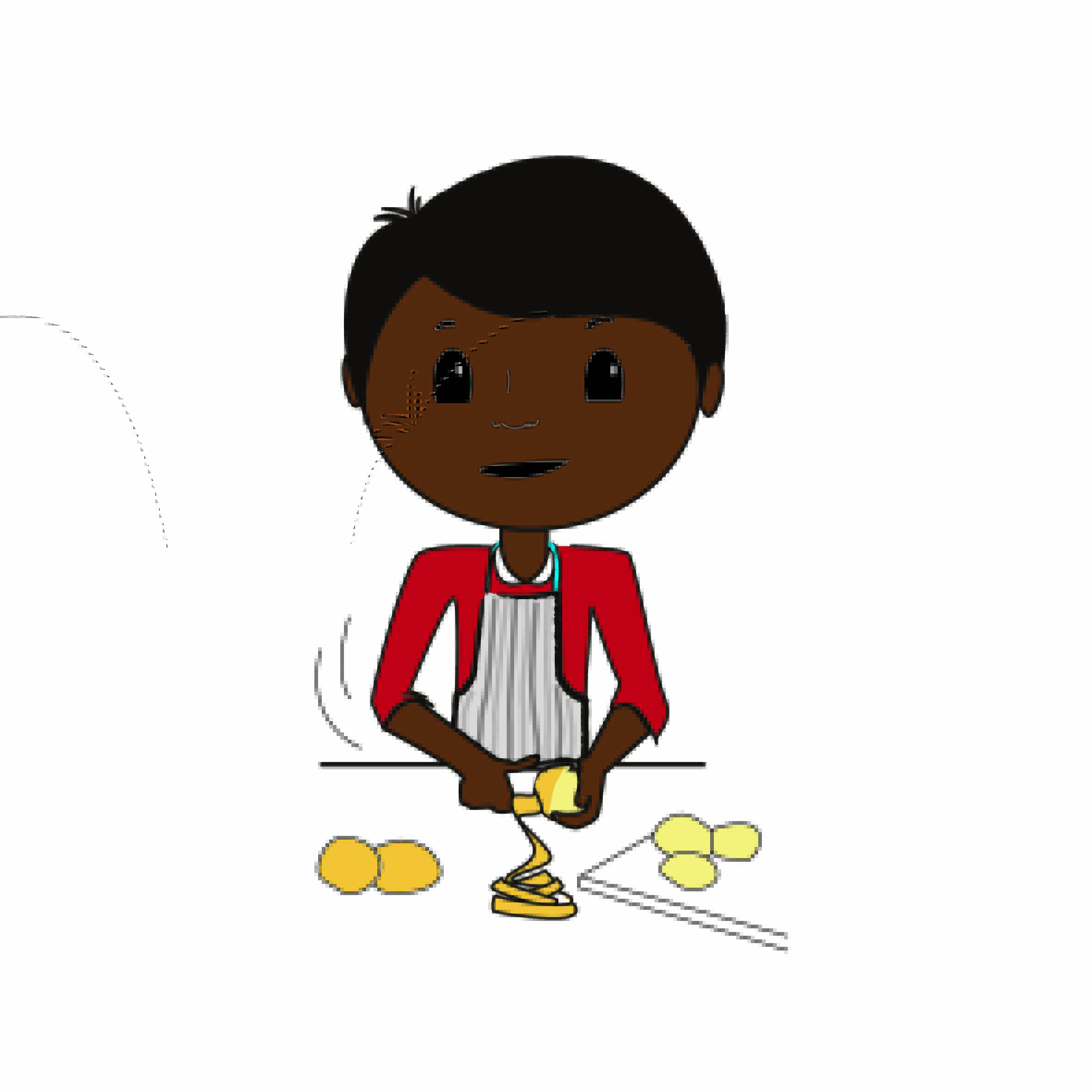 |
| --- | --- |
| A is not that good at peeling | B is really good at peeling |

Which are you MOST like?

□ I am a lot like A

□ I am a little like A

□ I am a bit like A and B

□ I am a little like B

□ I am a lot like B

Question 17


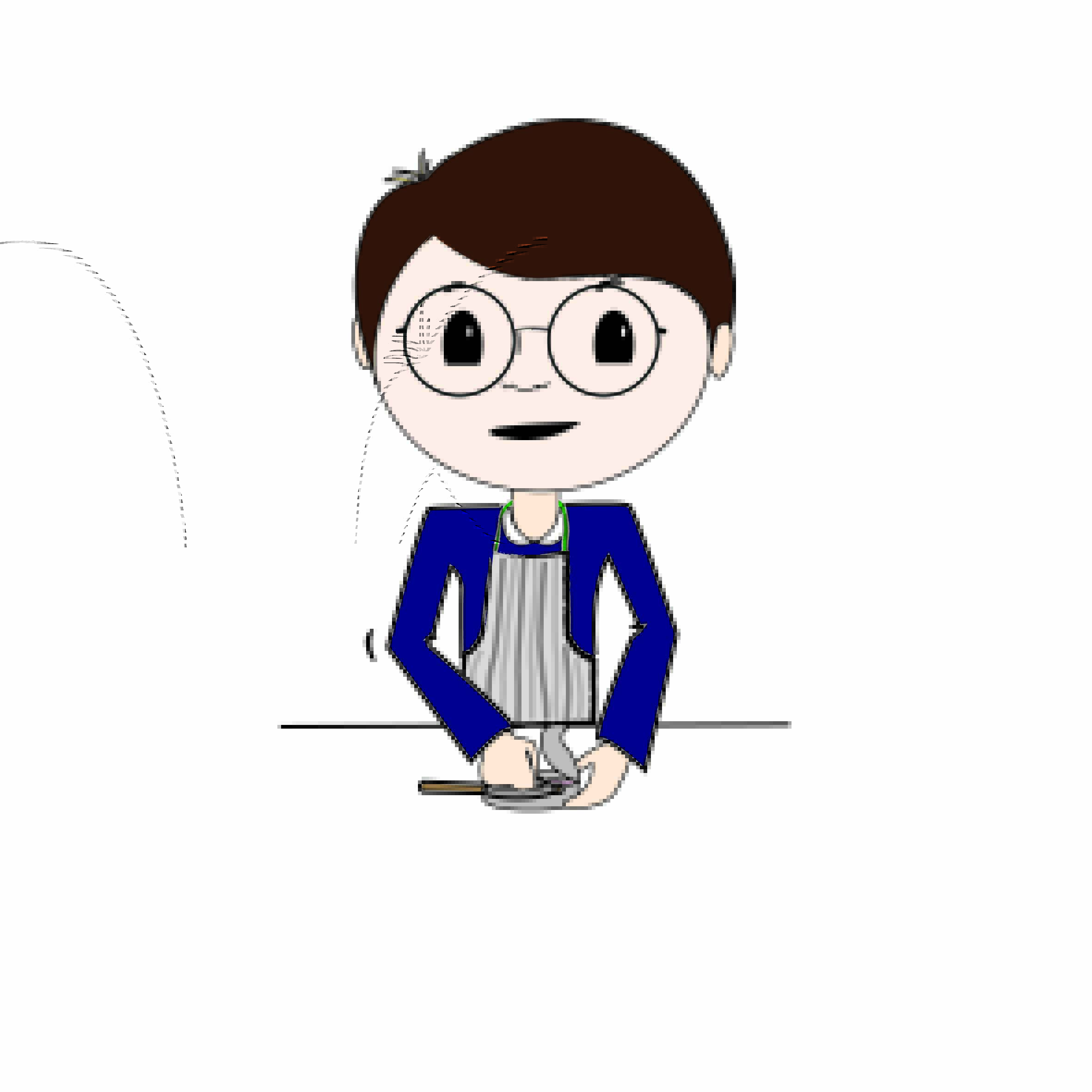


This child is using a tin opener. Do you do this?

□ Yes

□ No

Question 18

| 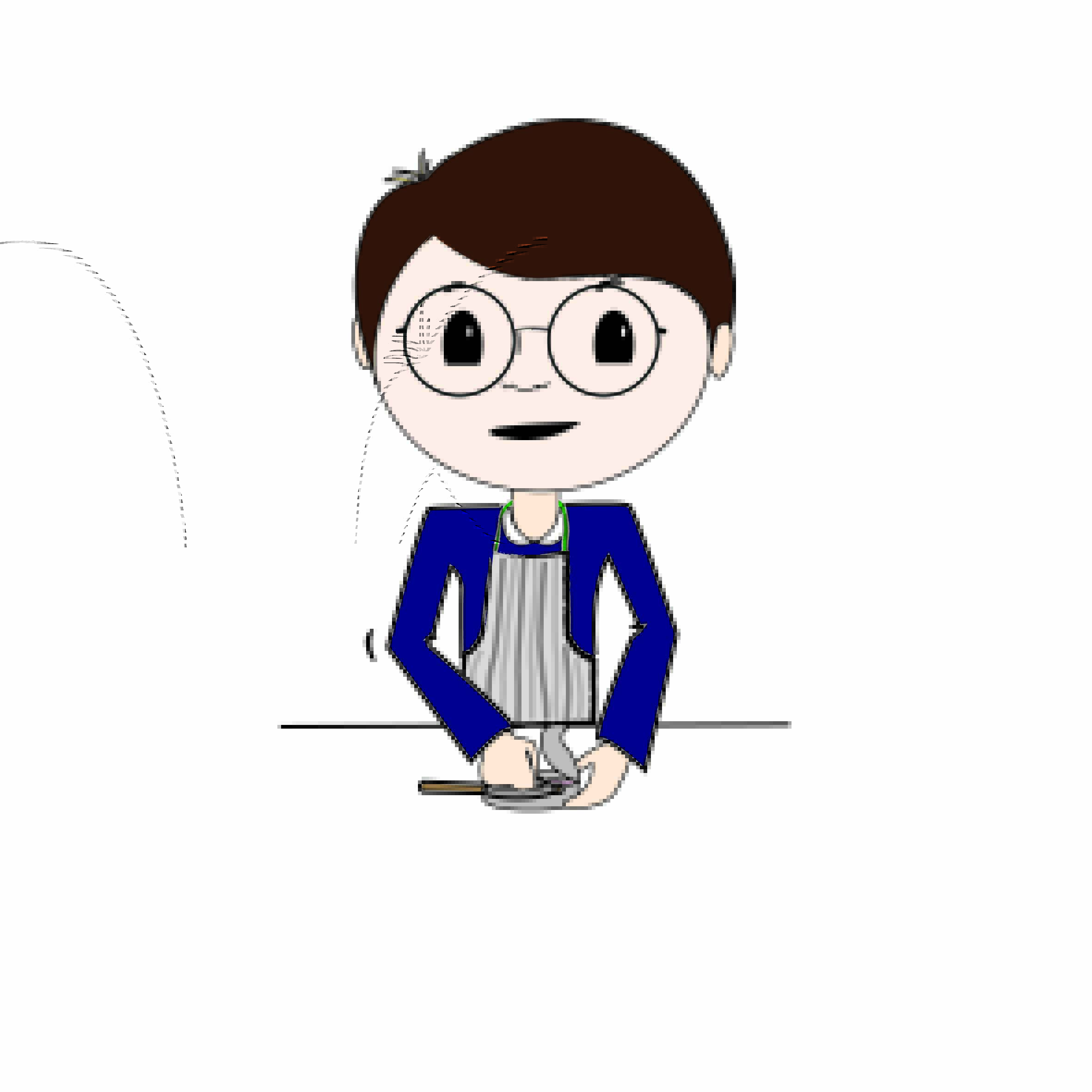 | 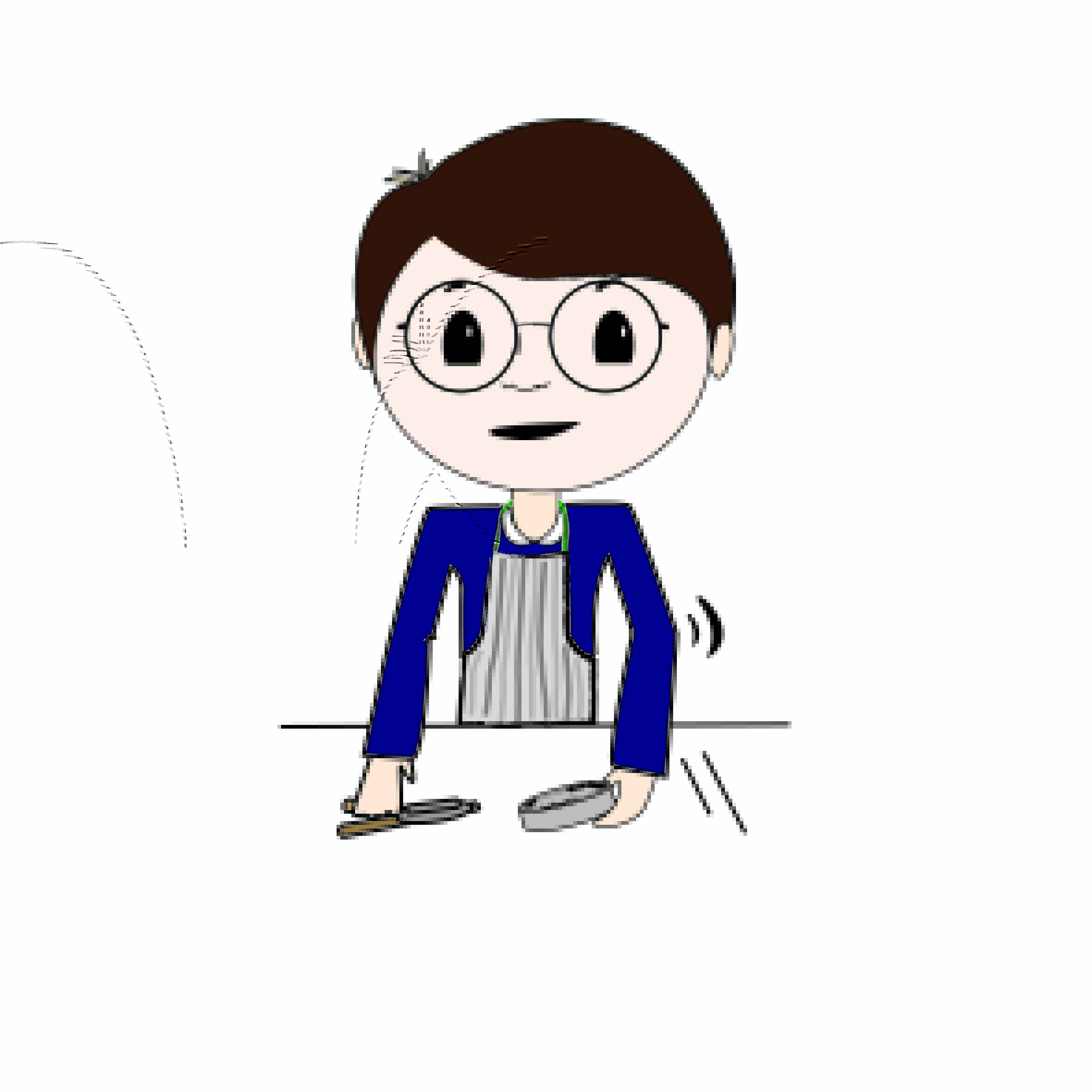 |
| --- | --- |
| A is really good at using a tin opener | B is not that good at using a tin opener |

Which are you MOST like?

□ I am a lot like A

□ I am a little like A

□ I am a bit like A and B

□ I am a little like B

□ I am a lot like B

Question 19


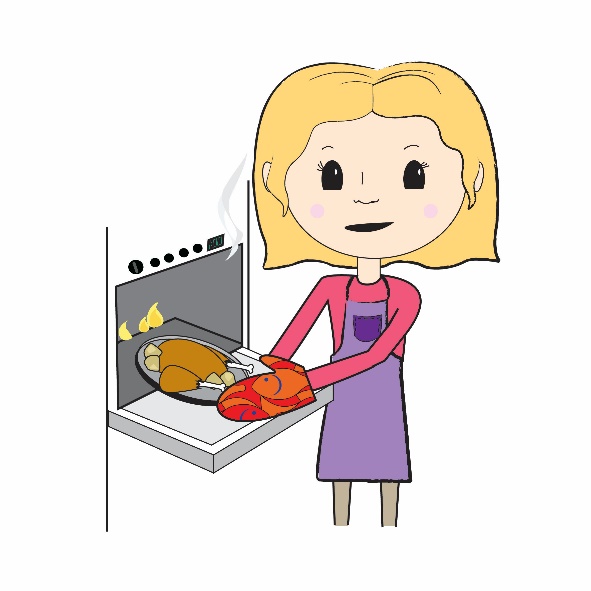


This child is using the oven. Do you do this?

□ Yes

□ No

Question 20

| 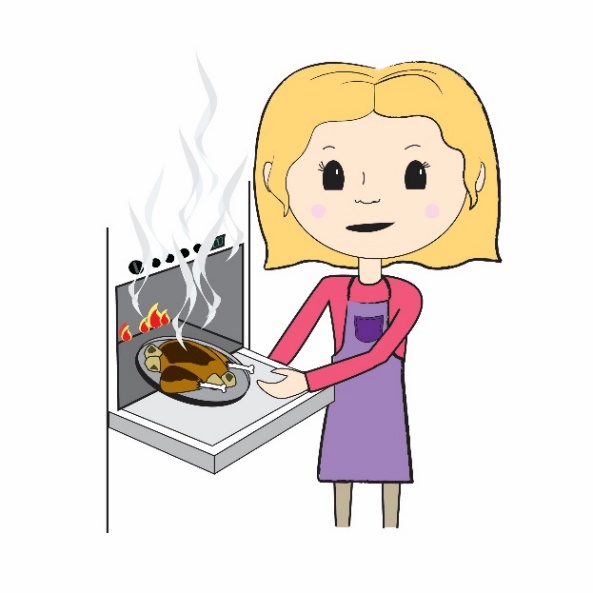 | 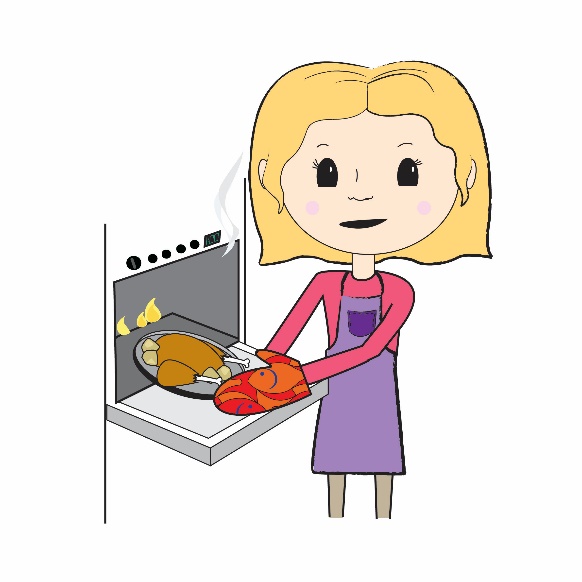 |
| --- | --- |
| A is not that good at using the oven | B is really good at using the oven |

Which are you MOST like?

□ I am a lot like A

□ I am a little like A

□ I am a bit like A and B

□ I am a little like B

□ I am a lot like B

Question 21


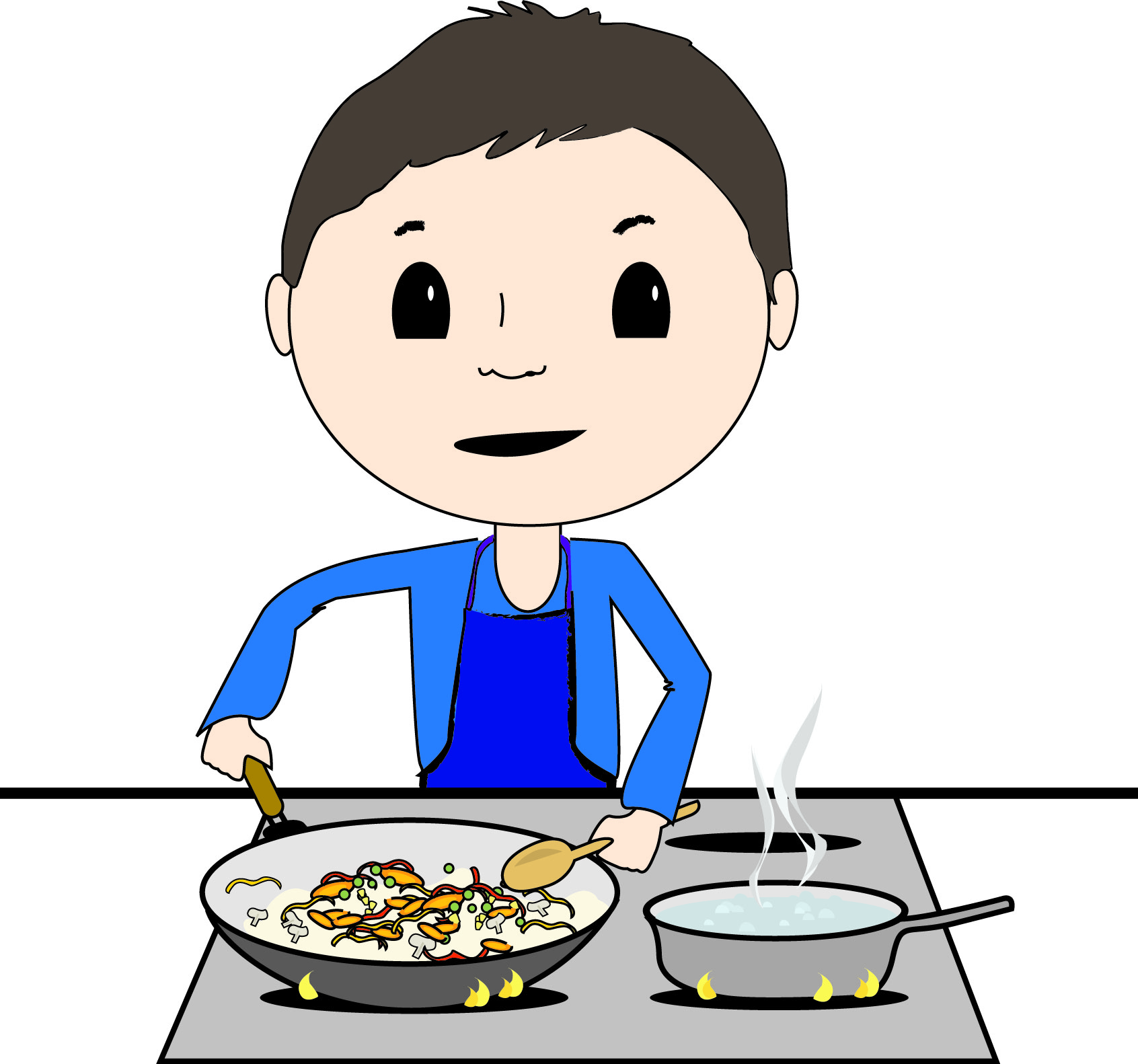


This child is using the stove/hob. Do you this?

□ Yes

□ No

Question 22

| 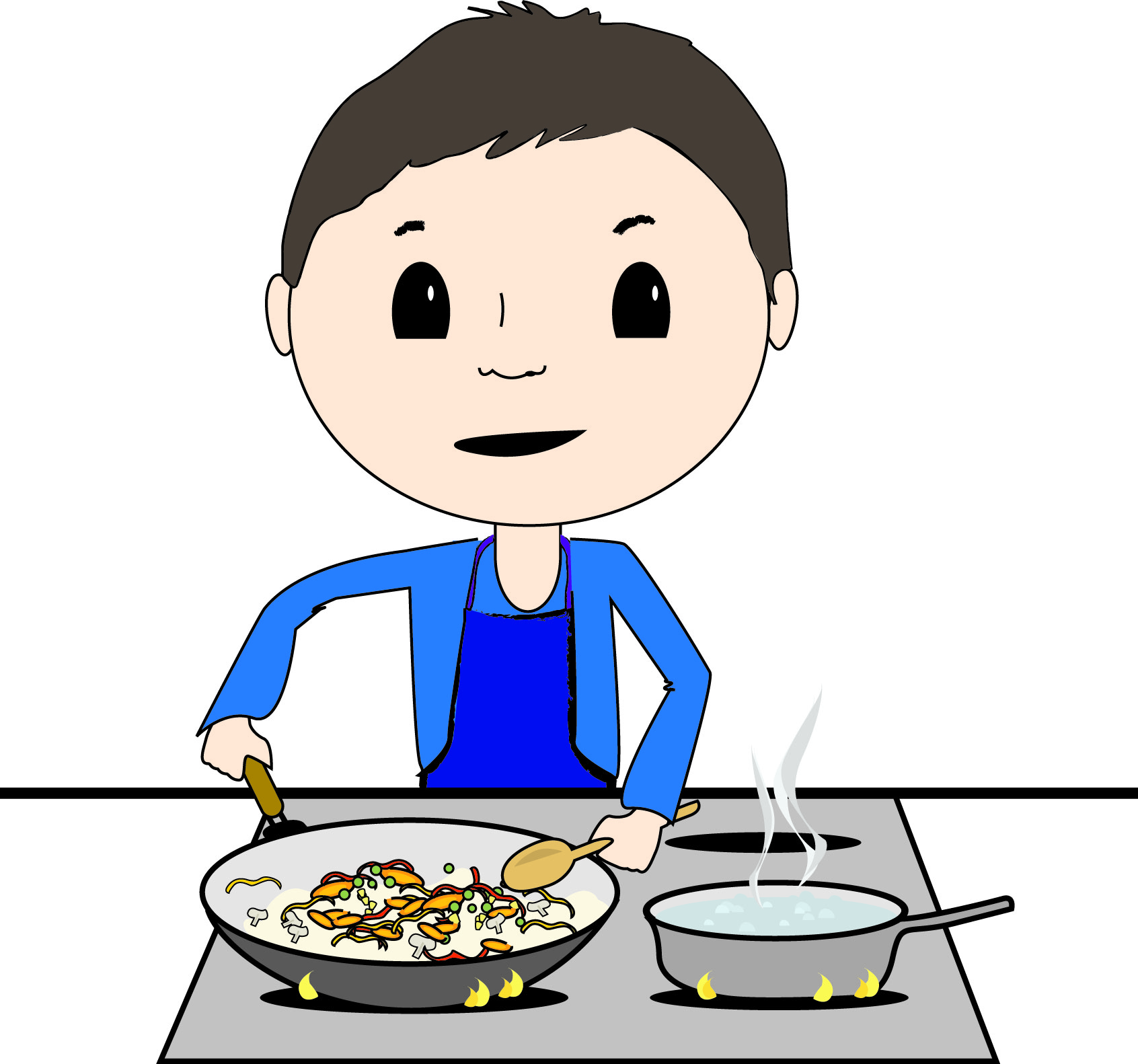 | 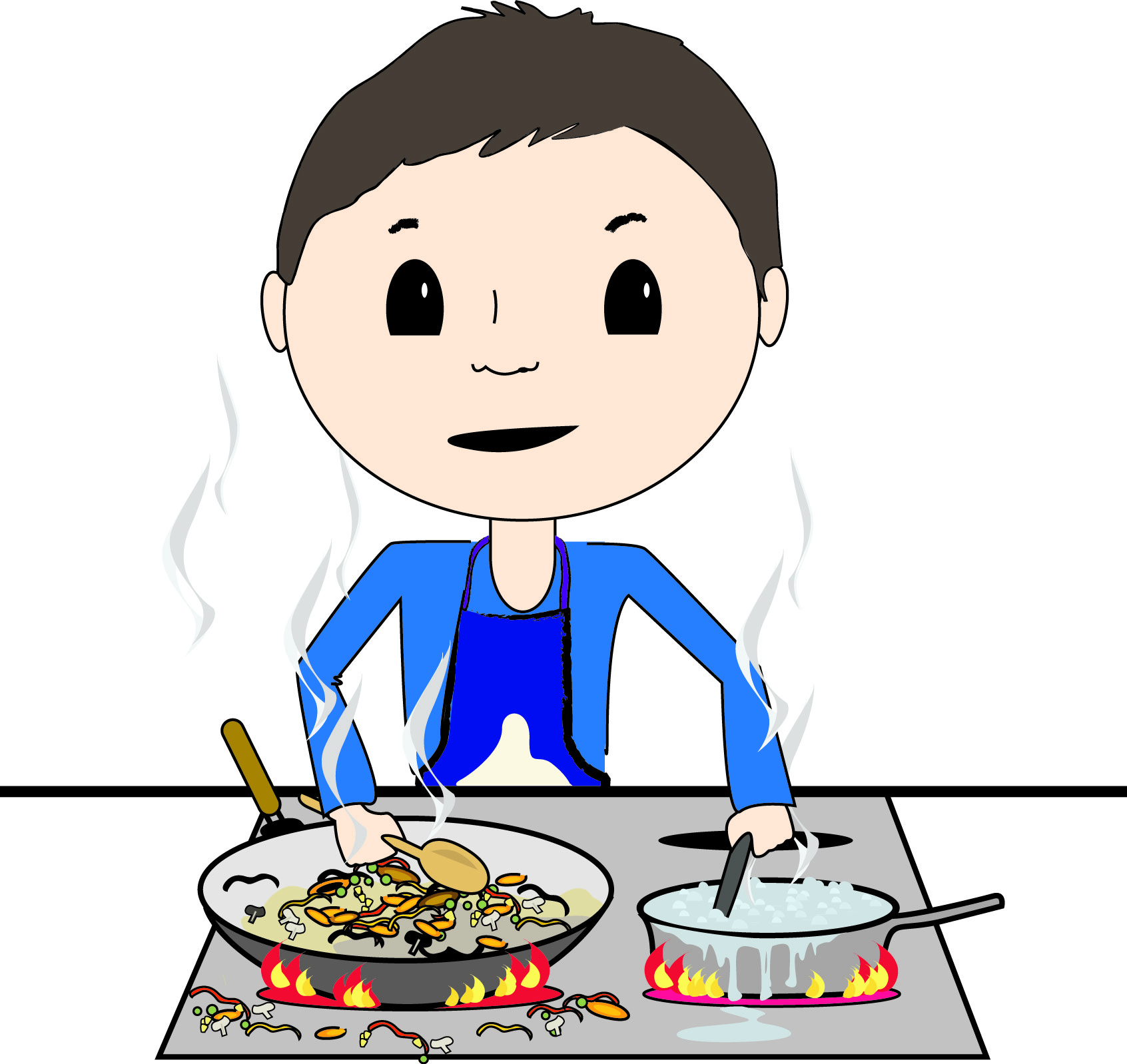 |
| --- | --- |
| A is really good at using the stove/hob | B is not that good at using the stove hob |

Which are you MOST like?

□ I am a lot like A

□ I am a little like A

□ I am a bit like A and B

□ I am a little like B

□ I am a lot like B

You have finished!

1. Dean, M., Issartel, J., Benson, T., McCloat, A., Mooney, E., McKernan, C., Dunne, L., Brennan, S. F., Moore, S. E., McCarthy, D., Woodside, J. V., & Lavelle, F. (2021). CooC11 and CooC7: the development and validation of age appropriate children’s perceived cooking competence measures. The International Journal of Behavioral Nutrition and Physical Activity, 18(1), 20–20. https://doi.org/10.1186/s12966-021-01089-9 [↑](#footnote-ref-1)
