# Supplementary material for: Evaluation of the school-based ‘PhunkyFoods’ intervention: a cluster randomised controlled trial in the UK
Source: Public Health Nutr. 2025 Apr 14;28(1):e86. doi: 10.1017/S1368980025000552 (PMC12100561; doi:10.1017/S1368980025000552)
Supplement: Vaughan et al. supplementary material 5 — Vaughan et al. supplementary material [file S1368980025000552sup005.docx]

## Appendix 4 Intervention Delivery Additional Information

Of the 13 intervention schools, 12 completed the staff training and 11 completed the health check and action planning activities. Five schools went beyond the first two ‘active ingredients’ and undertook a review of school policies on school food and packed lunches.

Eleven out of thirteen schools participated in the Phunky Ambassadors programme, which involved mentoring pupils to deliver key healthy lifestyle messages through peer-to-peer learning. Twelve out of 13 schools participated in whole school assemblies and pupil workshops on a range of topics relating to PhunkyFoods. Examples of whole school activities were: ‘food waste’, ‘drain your drinks’, ‘snack attack’ and ‘strive for 5’. For the ‘food waste’ topic in one school, year 5 (children aged 9 – 10 years) ambassadors were trained on food waste and delivered a presentation to the whole school via an assembly. The ambassadors then led a food waste audit across the whole school in one day and provided a summary report on their findings in another assembly. In another school, year 5 ambassadors delivered an assembly on ‘drain your drinks’ and the importance of good hydration, the comparisons of sugar content in certain drinks and what this means for health. The ‘strive for 5’ assembly was delivered by year 5 ambassadors for a whole school on the importance of eating 5 portions of fruit and vegetables per day and pupils were encouraged to “eat a rainbow” of different coloured fruits and vegetables. The ‘snack attack’ message was delivered in some schools in whole assemblies with ambassadors trained to deliver the key messages about the amount of sugar children should have as well as learning about recommended healthy snacks that could be eaten from the UK Eatwell Guide ^1^.

Twelve schools participated in the experiential curriculum classroom-based activities with healthy eating topics, for example ‘supercrunch’, ‘strive for 5’, ‘healthy lunches’ and cooking tasks linked to the religious education curriculum. In one school, year 5 ambassadors were involved in delivering a parent engagement session in a year 1 (children aged 5 – 6 years) class. This included an introduction to the Eatwell and Strive for 5 messages and demonstrating safe chopping techniques. The ‘healthy lunches’ classroom activity involved a recap on healthy lunches messages from assembly and demonstration of safe chopping skills. Children made a healthy bagel in the shape of a face snack using cream cheese and prepared vegetables. In one school, world curriculum topics were linked to cookery sessions in the classroom and this included making South Asian foods such as samosas, aloo chaat, ranoli fruit patterns and mango lassi. In these classroom sessions, ingredients were linked back to the UK Eatwell Guide and children developed their food preparation skills using the claw and bridge cutting technique^2^.

Seven schools participated in an afterschool cooking club delivered by the EDCs, most often this included parents. In one school the club ran for four weeks and different families attended each week. Families worked together to make pizza dough and chop ingredients to put on the pizzas. Another school had a cook club that ran for four weeks for year 5/6 children (aged 9 – 11 years), during which children developed their cutting, measuring, grating, peeling and kneading skills and followed recipes to make pizza, spring rolls, bread and orange shortbread. In one school six families attended four weekly sessions (involving 12 children and 10 adults each week) and made “dare devil” and super salmon dips with vegetable and pitta snacks, vegetable pizzas, bread rolls and tuna cous cous salad. At another school, seven families attended four weeks of cook club and made recipes to facilitate a wide range of different skills to be practiced: the bridge and claw safe cutting technique, weighing, measuring, mixing, peeling grating and kneading. For more information about the recipes, see the Supplementary Materials and the PhunkyFoods website.

1. England N. The Eatwell Guide. 2022 [updated 29 November 2022]; Available from: <https://www.nhs.uk/live-well/eat-well/food-guidelines-and-food-labels/the-eatwell-guide/>.

2. PhunkyFoods. Knife Skills: BRIDGE. YouTube2020.
